# The relationship between bipedalism and growth: A metric assessment in a documented modern skeletal collection (Certosa Collection, Bologna, Italy)

**DOI:** 10.1002/ajpa.24440

**Published:** 2021-11-16

**Authors:** Annalisa Pietrobelli, Damiano Marchi, Maria Giovanna Belcastro

**Affiliations:** ^1^ Department of Biological, Geological and Environmental Sciences Alma Mater Studiorum‐University of Bologna Bologna Italy; ^2^ Department of Biology University of Pisa Pisa Italy; ^3^ Evolutionary Studies Institute and Centre for Excellence in PalaeoSciences University of the Witwatersrand Johannesburg South Africa; ^4^ Natural History Museum of the University of Pisa Calci Italy

**Keywords:** fibula, linear geometry, motor skill development, prepubertal sex dimorphism, tibia

## Abstract

**Objectives:**

Long bone variations during growth are susceptible to the combined action of nutritional, hormonal, and genetic factors that may modulate the mechanical forces acting upon growing individuals as they progressively acquire a mature gait. In this work, we explore diaphyseal length and breadth variations of tibia and fibula during ontogeny (a) to test the presence of changes in relation to early toddling, and (b) to further our understanding of developmental patterns in relation to sex.

**Materials and Methods:**

Lengths, breadths, and indices were analyzed on right and left leg bones of 68 subadult individuals (Human Identified Skeletal Collection of the University of Bologna, Italy). Analyses included intersex and age classes (1, 0–1 year; 2, 1.1–3 years; 3, 3.1–6 years) comparisons, linear regressions with age and assessment of correlation among tibial and fibular measurements, as well as principal component analysis.

**Results:**

A significant difference emerged among age class 1 and the others. Age class 1 and 3 differ between them, while age class 2 overlaps with the others. No sex dimorphism was detected. All measurements were strongly correlated with age. Tibial and fibular measurements correlated with each other.

**Conclusions:**

Our results relate the progressive emergence of toddling attempts in growing individuals at the end of the first year of age. No significant sex differences were found, suggesting that tibial and fibula growth might diverge between sexes in later childhood. We provide quantitative data regarding tibial and fibular linear growth and its timing in a modern documented osteological sample from Italy.

## Graphical abstract

We provide quantitative data regarding tibial and fibular linear growth in relation to toddling and its timing in a modern documented osteological sample from Italy.

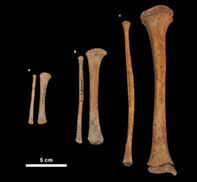

## 1 INTRODUCTION

Immature bone is subjected to continuous modeling due to variations in functional and biomechanical stresses that occur during ontogeny (Lieberman et al., 2001, 2003; Raab et al., 1990; Steinberg & Trueta, 1981). However, long bone diaphyseal shape and size variation during growth is influenced by the combined action of nutritional, hormonal, and genetic factors, that may modulate the mechanical forces acting upon growing individuals as they progressively acquire a mature gait (Gosman et al., 2011). Many studies have investigated how growth trajectories of long bones diaphyseal shape vary in relation to different locomotor behaviors and biomechanical requirements on subadult individuals (Cowgill et al., 2010; Cowgill & Johnston, 2018; Goldman et al., 2009; Gosman et al., 2011, 2013; Macdonald et al., 2006; Ruff, 1994, 2003a, 2003b; Ruff et al., 1994; Sumner, 1984; Sumner & Andriacchi, 1996).

Mechanical and structural properties of the femoral diaphysis in subadults revealed the influence of loading regimens during mobility on ontogenetic trajectories (Cowgill et al., 2010; Cowgill & Johnston, 2018). In particular, the authors analyzing ground reaction forces and diaphyseal cross‐sectional geometric (CSG) evidence in subadults individuals showed that femoral midshaft shape is correlated to load changes that happen during bipedal development during infancy: from a more rounded femoral midshaft produced by the higher mediolateral loads in the early stages of infancy to a more anteroposteriorly elongated one due to the progressively more anteroposterior‐oriented loads to which the femur is subjected as bipedal locomotion develops. Similar results have been obtained by Goldman et al. (2009), who analyzed both femoral midshaft CSG and histological properties. The authors also argued that the histological manifestations of cortical bone resorption and formation play a key role in diaphyseal shape changes, as cortical drift patterns emerge before any measurable change in the biomechanical properties of the midshaft femoral cross section. Other studies (Ruff, 2003a, 2003b) found early changes in the femoral and humeral strength proportions in subadults and interpreted them as the effect of the initiation of upright walking. In particular, comparing femoral and humeral growth patterns, Ruff (2003a) found a peak in growth velocity at mean age of 1.4 years, corresponding to the initiation of bipedal walking. A similar peak was found slightly earlier with a subsequent steep decline interpreted as the result of the shift from crawling to independent walking and therefore changing the humeral loading regimen (Ruff, 2003b).

Regarding the leg, while the body of evidence on the structural and biomechanical properties on the tibiofibular complex in adults is progressively increasing (Auerbach et al., 2017; Marchi & Shaw, 2011; Rantalainen et al., 2010, 2014; Tümer et al., 2019), scarce information is available for subadult individuals, with analyses focusing mostly on the tibia (Gosman et al., 2013; Hubbell et al., 2011). The importance of considering leg bones together (and not the tibia alone) to better understand load distribution in the distal segment of the lower limb has been previously stressed in anthropological and biomechanical studies (Funk et al., 2004, 2007; McNeil et al., 2009; Scott et al., 2007). In particular, some studies brought attention to the functional role of the fibula in transmitting to the foot a portion of the mechanical load encountered during gait by the leg, which varies between 5% and 19% depending on ankle position (Funk et al., 2004; Goh et al., 1992; Lambert, 1971; Takebe et al., 1984). Moreover, recent research on the diaphyseal CSG properties of the fibula allowed the association of fibular structure to diverse mobility patterns in modern humans (Auerbach et al., 2017; Hagihara & Nara, 2016; Lüscher et al., 2019; Marchi et al., 2011; Marchi & Shaw, 2011; Sparacello et al., 2014), great apes (Marchi, 2005, 2007), and non‐hominoid primates (Marchi, 2015b), with further application in paleoanthropology and the origins of bipedal locomotion (Marchi, 2015a; Marchi et al., 2019).

The role of the tibia during growth and the onset of bipedal walking has been investigated by Ireland et al. (2014), who found an association between the timing of unsupported walking (~15 months) and tibiae greater bone mass, cortical bone area, pericortical circumference and polar moment of inertia, even when sex and body size were taken into account. Other studies observed a shift of midshaft cross‐sectional shape from relatively circular in early childhood to more anteroposteriorly orientated in early puberty (Gosman et al., 2013; Hubbell et al., 2011). Finally, Cowgill and Johnston (2018) proposed an evaluation of humeral to tibial, and femoral to tibial strength ratio to identify a “walking peak” in a large Holocene subadult skeletal sample. The authors found a more defined peak of humeral to tibial strength at the age corresponding to children shifting from crawling to walking and interpreted the result as the effect of the limited load to which the tibia is subjected during crawling compared to the femur and to the more dramatic biomechanical transition experienced by the tibia during this walking pattern transition.

Patterns of sex and age variations in relation to diaphyseal lengths and breadths in subadults have been explored by traditional morphometric studies on tibial and fibular diaphyses. In general, no sex‐related difference is found for tibial length and breadth until 15 years of age (Cardoso et al., 2014; López‐Costas et al., 2012). On the other hand, Humphrey (1998) found that tibial and fibular breadths may slightly diverge between sexes since earlier in childhood (4.2–5.3 years for tibial diameters; 2.3–11.2 years for fibular diameters). Regarding age variations, the positive relationship between age and long bone diaphyseal length, epiphyseal and metaphyseal widths and breadths has been observed in many different populations and used to provide specific standards for age estimation in subadult individuals (Black & Scheuer, 1996; Cardoso et al., 2014, 2017; López‐Costas et al., 2012; Maresh, 1943, 1955, 1970; Primeau et al., 2012, 2016; Rissech et al., 2008, 2013; Stull et al., 2014, 2017; Tsai et al., 2016).

In this work, we perform a quantitative traditional morphometrics study of tibia and fibula diaphyses of subadult individuals (*n* = 68) aging 0–6 years, belonging to the Human Identified Skeletal Collection of the University of Bologna (Belcastro et al., 2017). The aim of this research is to better characterize linear and geometric changes in the diaphyses of tibia and fibula during growth in relation to biological sex and age, providing new research data and contributing to the understanding of the developmental patterns concerning sex and age. Based on previous literature, we will test the following hypotheses:

- We hypothesize for both tibia and fibula a shift from subcircular symmetric outline of the diaphysis (i.e., similar sagittal and transverse diameters along the whole shaft) in younger individuals toward a more anteroposterior‐oriented outline (i.e., relatively greater anteroposterior diameters along the whole shaft) in older individuals in relation to the onset of bipedal locomotion (Cowgill et al., 2010; Cowgill & Johnston, 2018; Goldman et al., 2009; Gosman et al., 2011, 2013). Moreover, we expect to find a similar longitudinal growth pace (i.e., diaphyseal length) for both bone diaphyses and a positive correlation among tibial and fibular metrics, given the two bone proportionate interaction that is crucial for the normal development of the lower leg (Beals & Skyhar, 1984).
- We hypothesize little to no sex dimorphism in diaphyseal size and shape and a strong relationship with age for all measurements for the two bones, which may proceed according to growth spurts (Cardoso et al., 2014; López‐Costas et al., 2012)

## 2 MATERIALS AND METHODS

**FIGURE 1:**
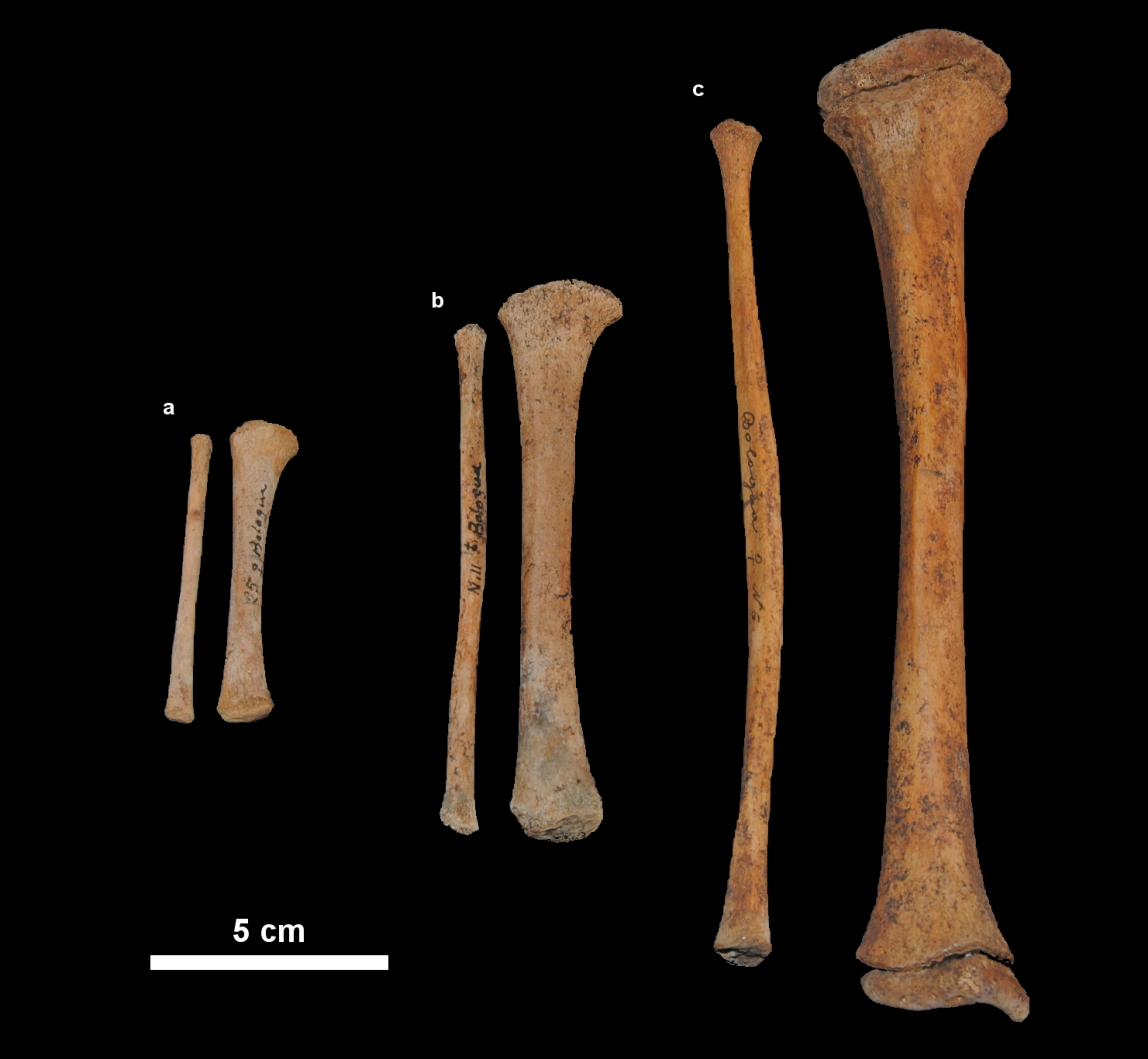
Right tibiae and fibulae belonging to three individuals from the human‐identified skeletal collection of the University of Bologna, representing different age classes. Tibiae are displayed in anterior view, while fibulae are displayed in anterolateral view. (a) Age class 1: BO25, female, 9 days old; (b) age class 2: BO11, male, 1 year and 3 months old; (c) age class 3: BO6, female, 5 years and 10 months old

**FIGURE 2:**
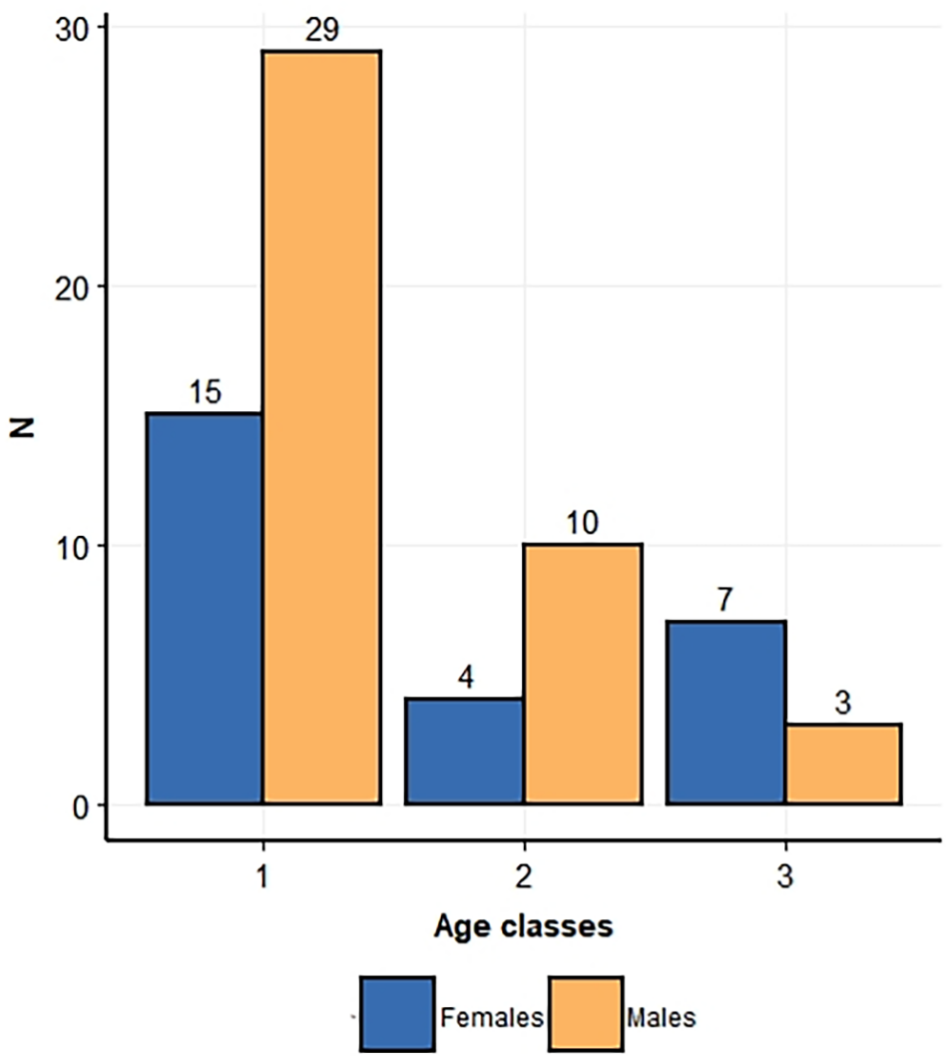
Barplot representing sample composition, with subdivision by sex and age classes 1 (from 0 to 1 year of age), 2 (from 1.1 to 3 years of age), and 3 (from 3.1 to 6 years of age)

The sample analyzed in this study refers to right and left tibiae and fibulae of 68 subadult individuals belonging to the Human Identified Skeletal Collection of the University of Bologna. This collection, housed at the Museum of Anthropology of University of Bologna, was put together by Fabio Frassetto (1876–1953) and Elsa Graffi Benassi (1901–2000) in the first half of the 20th century, consisting of cemetery exhumations carried out between the late 19th and early 20th centuries (Belcastro et al., 2017). The analyzed sample includes both males and females spanning 0–6 years of age (Table 1; Figures 1 and 2).

**TABLE 1:** Sample composition by sex and age classes

Age class	Females	Males	Total
Age class 1	15	29	44
Age class 2	4	10	14
Age class 3	7	3	10
Total	26	42	68

*Note*: Age class 1 = 0–1 years of age; age class 2 = 1.1–3 years of age; age class 3 = 3.1–6 years of age.

This identified skeletal collection, with a total of 126 subadult individuals, includes information on the sex, age‐at‐death, and social status of each individual, ensuring exhaustive and punctual biological parameters on each individual profile. Sources for these parameters include cemetery and hospital records, as well as anagraphic data (e.g., birth certificates and residence certificates from public archives). All specimens had unfused proximal and distal epiphyses on both tibia and fibula. Individuals with documented skeletal pathologies such as metabolic disease or trauma were not included in this study (Tanganelli, n.d.). Moreover, tibiae and fibulae with postmortem damage or other taphonomic alterations were excluded from analysis.

### 2.1 Age classes

Age class subdivision was designed with reference to medical literature, considering different stages of locomotor behavior in growing children progressively acquiring bipedal locomotion according to specific patterns and timing. Age class 1 includes individuals from 0 to 1 year of age: by the end of this stage, children normally develop an immature toddling gait. Starting from birth, children usually progress to toddling through an early phase (up to 6 months of age), in which weight‐bearing on lower limbs is absent, characterized by precursory locomotor movements such as supine kicking and supported sitting (Thelen et al., 1984; Thelen & Fisher, 1982), as well as postural control in pronation, including chin and torso holding and rolling with upper limb support (Adolph & Joh, 2007; Bly, 1994; Swan et al., 2020). Following a brief phase (up to 8 months of age) of dependent/independent crawling and scooting, infants usually acquire a standing position and begin cruising toward the end of first year of age, at first while holding on to objects or caregivers for support and ultimately to independent toddling (Adolph et al., 1998; Bly, 1994).

Age class 2 includes individuals from 1.1 to 3 years of age: during this phase, independent toddling is at its early stages, as the product of a gradual maturation of the locomotor pattern during the period of supported locomotion, ultimately leading to unsupported plantigrade walking at a slow, irregular pace (Hallemans, De Clercq, & Aerts, 2006). At this stage the flexed position of the hip and knee lead to a dominance of plantarflexing movements at the ankle, while the upper limbs are abducted with a slightly flexed forearm (Forssberg, 1985; Hallemans et al., 2003; Hallemans, De Clercq, & Aerts, 2006; Hallemans, De Clercq, Dongen, & Aerts, 2006; McGraw, 1940, 1945; Stout, 2004; Swan et al., 2020). As the torso leans forward, the pelvis is forced to tilt mediolaterally during the swing phase of the stride, since the flexed hip contralateral to the standing leg induces the swinging leg to elevate (Hallemans et al., 2004). By the end of this phase, children usually engage in a more mature toddling pattern, with improved gait, longer steps, and a loading pattern of an initial heel‐strike (Adolph et al., 2003; Hallemans, De Clercq, Dongen, & Aerts, 2006; Ivanenko et al., 2004; Swan et al., 2020; Zeininger et al., 2018).

Age class 3 includes individuals from 3.1 to 6 years of age: this phase spans late toddling to mature bipedal gait. At the beginning of this phase, children usually begin their stride with the center of pressure under the calcaneus, consistent with the pattern of initial heel‐strike seen in adults (Zeininger et al., 2018). Afterwards, mature bipedal gait is progressively acquired: steps become longer, narrower, straighter, and more consistent with an adult walking gait, as the result of an increased stability produced by elevated femoral bicondylar angle that adducts the knee (Swan et al., 2020; Tardieu & Trinkaus, 1994).

### 2.2 Skeletal leg development during growth (0–6 years of age)

#### 2.2.1 Age class 1 (0–1 year)

Primary ossification centers for tibial shaft appear at 7–8 weeks in utero. At birth, tibial shaft is arched posteriorly in the proximal third and straight in the distal two‐thirds, while borders are usually blunt and less marked, with an evident nutrient foramen posteriorly. The perinatal fibula appears straight and slender, with rounded or angled outline in the proximal half and flattened mediolaterally in the distal half (Figure 1a). Its primary ossification center usually appears around 8 weeks in utero but does not begin ossification until the end of fetal period (O'Rahilly & Gardner, 1975). Posterolaterally, the subcutaneous triangular surface (STS) is often porotic‐looking, while at the distal end of the medial surface, where the inferior transverse part of the posterior tibiofibular ligament inserts, appears as a roughened triangle.

By 6 weeks after birth, tibial proximal secondary center appears. During the first few months after birth, the tuberosity develops distally to the main proximal tibial growth plate, followed by tibial distal secondary centers around 3–10 months of age (Schaefer et al., 2009; Scheuer & Black, 2000). Around the age of 1, when toddlers normally start to walk, the foot skeleton is formed by partially ossified centers, connected by soft tissue, with no visible longitudinal arch, whose bony structure only starts developing approximately at the end of this phase (Hallemans, De Clercq, Dongen, & Aerts, 2006). In the meantime, tibial shaft, despite certain variations, usually rotates 5° laterally (tibial shaft rotates another 10° by mid‐childhood and in older children and adult lateral torsion degree may reach 14°, Staheli & Engel, 1972). Tibial distal epiphysis starts to ossify, in parallel to the appearance and consequent ossification of the fibular distal epiphysis (Hoerr et al., 1962; Schaefer et al., 2009; Scheuer & Black, 2000).

#### 2.2.2 Age class 2 (1.1–3 years)

During the second year of age, the proximal portion of the fibular shaft is more flared and consequently the neck also becomes more evident (Figure 1b). The STS is also well marked, with a flat distal metaphyseal surface. The proximal tibial epiphysis progresses its osseous expansion and appears flattened inferiorly and extended superiorly toward the tibial spines (Schaefer et al., 2009; Scheuer & Black, 2000).

#### 2.2.3 Age class 3 (3.1–6 years)

Around 3–4 years of age, the tibial proximal epiphysis is shaped as an elongated nodule, rounded superiorly, with a pitted surface. The metaphyseal surface is flattened, with a roughly oval outline. Ossification of the tibial proximal epiphysis extends into the intercondylar region and the tubercles by 6–7 years of age. The relative articular surface is smooth, and the condyles have reached their characteristic adult morphology. Regarding the fibula, at 4 years of age in girls and 5 in boys, ossification of the fibular proximal epiphysis begins, but the timing is variable (Hoerr et al., 1962). Proximal fibular epiphysis has completed ossification and presents a rounded superior border, in level with the tibial growth plate, but does not assume adult appearance until late childhood (Scheuer & Black, 2000; Schaefer et al., 2009; Figure 1c).

The tibial distal epiphysis becomes recognizable at 3–4 years of age, shaped as an oval disc, with a projecting beak on the anteromedial aspect of the metaphyseal surface. By 3–5 years of age, the tibial medial malleolus starts to ossify. Growth is rapid, in keeping with that of the foot and by 5 years in girls and 6.5 years in boys the distal epiphyseal and metaphyseal widths are equal. Parallelly, at around 3 years of age the growth plate of the fibular distal epiphysis is at level with the tibiotalar articular surface, as a further response to the biomechanical necessities of bipedal walking. The bony fibular distal epiphysis is usually recognizable by this time and is an irregular nodule of bone with a flat metaphyseal surface (Schaefer et al., 2009; Scheuer & Black, 2000). By 6–7 years of age, the shaft of the fibula, similarly to the shaft of the tibia whose soleal line usually appears by this time as a well‐distinguishable porotic fossa or ridge (Belcastro et al., 2020), has achieved adult morphology and the main borders and surfaces can usually be identified, while the distal fibular epiphysis is almost completely ossified, with a well‐defined malleolar fossa (Schaefer et al., 2009; Scheuer & Black, 2000).

### 2.3 Anthropometric measurements

**FIGURE 3:**
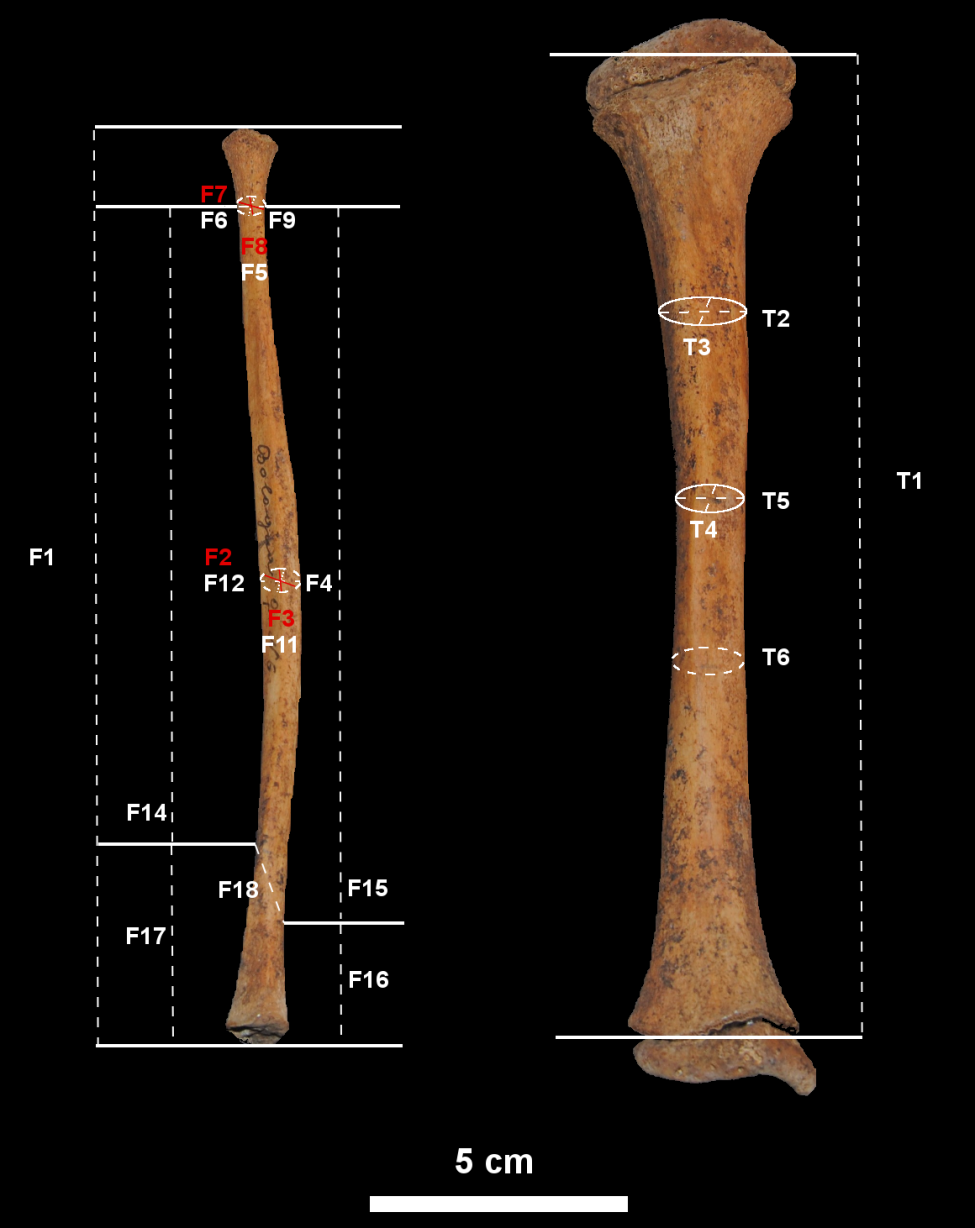
Tibial and fibular measurements, obtained by anthropological literature selected for this study and specifically designed for this study. See Tables 2 and 3 for measurement explanation

Anthropometric measurements were acquired using an osteometric board, a sliding digital caliper (Mitutoyo Digimatic caliper; resolution: 0.01 mm) and an anthropometric tape measure (Holtain LTD Harpenden Anthropometric tape). Table 2 and Figure 3 present the anthropometric measurements on the tibiae selected for this study. Table 3 and Figure 3 show the anthropometric measurements on the fibulae selected for this study.

**TABLE 2:** Tibial measurements and indices, obtained by anthropological literature, selected and modified for this study

Nr.	Definition	Description	References
T1	Maximum tibial length	Distance from the most prominent point on the proximal metaphyseal plate to the most prominent point on the distal metaphyseal plate	Modified after Martin and Saller (1957), #1
T2	Tibial sagittal shaft diameter at nutrient foramen	The greater distance from anterior border to the posterior surface at the level of the nutrient foramen	Martin (1928), 1050, #8a; Buikstra e Ubelaker (1994): 83, #72
T3	Tibial transverse shaft diameter at nutrient foramen	The maximum mediolateral (i.e., coronal) dimension of the shaft at the level of the nutrient foramen	Martin (1928), 1050, #9a; Buikstra e Ubelaker (1994): 83, #73
T4	Tibial sagittal midshaft diameter	Anteroposterior diameter at 50% of tibial length, from the anterior crest to the posterior surface	Martin (1928), 1050, #8
T5	Tibial transverse midshaft diameter	Mediolateral (i.e., coronal) diameter at 50% of tibial length	Martin (1928), 1050, #9
T6	Minimum shaft circumference	Minimum circumference, usually at the inferior third of tibial length	Krogman and Işcan (1986)
T7	Tibial midshaft shape index	(T5/T4) × 100	Martin and Saller (1957)

**TABLE 3:** Fibular measurements and indices, both obtained by anthropological literature, and specifically designed for this study

Nr.	Definition	Description	References
F1	Maximum fibular length	Distance from the most prominent point on the proximal metaphyseal plate to the most prominent point on the distal metaphyseal plate	Modified after Martin and Saller (1957), #1
F2	Fibular maximum diameter at midshaft	The greatest diameter of shaft at 50% of fibular length	Martin (1928): 1052, #2; Buikstra e Ubelaker (1994): 84, #76
F3	Fibular minimum diameter at midshaft	The minimum diameter of shaft at 50% of fibular length	Martin (1928): 1052, #3; Buisktra e Ubelaker (1994): 84, #77
F4	Circumference at midshaft	The minimum circumference of shaft at 50% of fibular length	Martin (1928): 1053, #4
F5	Sagittal diameter at neck	Distance form anterior border to posterior surface at fibular neck	Developed by DM
F6	Transverse diameter at neck	Distance from medial to lateral surfaces at fibular neck (i.e., coronal diameter)	Developed by DM
F7	Maximum diameter at neck	The greatest dimension at neck, usually found along the sagittal plane	Developed by DM
F8	Minimum diameter at neck	The shortest dimension at neck, usually found along the transverse plane	Developed by DM
F9	Circumference at neck	The minimum circumference at fibular neck	Developed by DM
F10	Fibular neck shape index	(F6/F5) × 100	Developed by DM
F11	Sagittal diameter at midshaft	Distance form anterior border to posterior surface at fibular midshaft	Developed by DM
F12	Transverse diameter at midshaft	Distance from medial to lateral surfaces at fibular midshaft (i.e., coronal diameter)	Developed by DM
F13	Fibular midshaft shape index	(F12/F11) × 100	Developed by DM
F14	Distance from neck to STS	Linear distance along the anterior border from fibular neck to the most proximal point of the subcutaneous triangular surface (STS)	Developed by DM
F15	Distance from neck to ILA	Linear distance along the medial surface from fibular neck to the most proximal point of the interosseous tibiofibular ligament attachment (ILA)	Developed by DM
F16	ILA length	Linear distance from the most proximal to most distal point of the ILA	Developed by DM
F17	STS length	Maximum distance from the most proximal to the most distal point of the STS	Developed by DM
F18	Distance from STS to ILA	Longitudinal distance from the most proximal point of the STS to the most proximal point of the ILA	Developed by DM
F19	STS‐ILA index	(F18/F15) × 100	Developed by DM
F20	STS index	(F17/F1) × 100	Developed by DM
F21	ILA index	(F16/F1) × 100	Developed by DM

Abbreviations: ILA, interosseous ligament attachment; STS, subcutaneous triangular surface.

### 2.4 Statistical analyses

All statistical analyses were carried out in RStudio (version 4.0.0 “Arbor Day,” R Core Team, 2020). Missing data were replaced with each variable mean value. To evaluate possible asymmetry between the left and the right side, a subsample (*N* = 30) was selected and a *t‐test* or a *Wilcoxon* test (McDonald, 2014) was carried out depending on each variable distribution. Analysis on the whole sample (*N* = 68) was accordingly performed. Normality distribution was assessed by *Shapiro–Wilk normality* test (Shapiro & Wilk, 1965). Descriptive statistics (mean, standard deviation, minimum and maximum values, and interquartile range) were then calculated for each variable on the whole sample and by sex and age class. For each variable, we assessed the presence of a linear correlation with age and calculated both a linear regression model and a LOESS fitted polynomial regression, with 95% confidence intervals and a smoothing value set at 0.6 (McDonald, 2014). This smoothing value was selected since it produced the best‐fitting curves, whereas lower values tended to excessively capture the random error in the data generated by outliers (Cleveland & Devlin, 1988). The *Kruskal–Wallis* test (Kruskal & Wallis, 1952) was used to evaluate possible differences among sexes and age classes and pairwise comparisons were performed using the *Dunn post‐hoc test* (Dunn, 1964). The correlation between tibiae and fibulae measurements was assessed by calculating linear regression models between homologous measurements (maximal length, sagittal midshaft diameter, transverse midshaft diameter, midshaft circumference, midshaft shape index) on the two leg bones. Finally, a principal component analysis (PCA) was carried out by computing a variance–covariance matrix, to explore data variance among sexes and age classes, utilizing the function *prcomp ()* that by defaults centers the data.

## 3 RESULTS

Analyses showed no significant difference (*p* < 0.05) between left and right side of both the tibia and fibula. Therefore, in the analyses we considered measurements taken on the right side, occasionally replaced by measurements of the left side if the former was absent. The Shapiro–Wilk normality test revealed that data were not normally distributed therefore for the following analyses we adopted nonparametric tests.

Tables 4 and 5 and Figure S1 present descriptive statistics and boxplots of linear measurements and shape indices for the tibia for the whole sample and by sex and age 1 and 2. Tables 6 and 7 and Figure S2 show descriptive statistics and boxplots of linear measurements and shape indices for the fibula for the whole sample and by sex and age.

**TABLE 4:** Descriptive statistics (mean, standard deviation, max–min values, interquartile ranges) for the tibia, considering the whole sample

	Mean (*SD*)	Min–max	1Qrt–3Qrt
Maximum tibial length	91.15 (39.15)	36.06–186.00	59.35–117.50
Tibial sagittal shaft diameter at nutrient foramen	9.52 (3.52)	3.60–17.39	6.50–11.97
Tibial transverse shaft diameter at nutrient foramen	9.15 (3.61)	3.98–18.68	6.00–12.13
Tibial sagittal midshaft diameter	8.38 (3.13)	3.42–14.74	5.61–10.90
Tibial transverse midshaft diameter	7.64 (2.85)	3.54–13.19	5.33–9.80
Minimum shaft circumference	27.60 (9.29)	14.00–47.00	19.50–36.00
Tibial midshaft shape index	91.78 (8.48)	78.56–112.71	85.40–96.22

**TABLE 5:** Descriptive statistics (mean, standard deviation, max–min values, interquartile ranges) for the tibia, considering age classes and sex groups

Age class 1	Males			Females		
	Media (*SD*)	Min–max	1Qrt–3Qrt	Media (*SD*)	Min–max	1Qrt–3Qrt
Maximum tibial length	64.43 (15.43)	40.53–109.00	56.98–65.20	72.97 (21.76)	36.06–118.00	59.32–90.13
Tibial sagittal shaft diameter at nutrient foramen	7.31 (1.96)	4.23–12.76	6.09–8.28	8.07 (2.57)	3.60–11.85	6.23–10.91
Tibial transverse shaft diameter at nutrient foramen	6.71 (1.66)	4.53–11.44	5.77–7.16	7.41 (2.04)	3.98–11.12	5.97–8.94
Tibial sagittal midshaft diameter	6.41 (1.81)	4.23–11.41	5.19–6.89	6.90 (2.15)	3.42–9.97	5.39–8.88
Tibial transverse midshaft diameter	5.68 (1.33)	3.83–9.01	4.71–6.09	6.42 (1.84)	3.54–9.55	5.27–7.36
Minimum shaft circumference	21.48 (5.12)	14.00–35.00	18.00–23.00	23.73 (6.60)	14.00–36.00	19.00–28.00
Tibial midshaft shape index	94.43 (6.90)	78.97–107.64	84.48–93.58	89.64 (9.83)	78.56–112.71	89.26–99.77

Age class 2	Males			Females		
	Media (*SD*)	Min–max	1Qrt–3Qrt	Media (*SD*)	Min–max	1Qrt–3Qrt
Maximum tibial length	117.64 (21.81)	66.43–145.00	111.25–130.00	120.00 (4.97)	115.00–126.00	116.50–123.00
Tibial sagittal shaft diameter at nutrient foramen	11.62 (2.11)	7.43–14.34	10.68–12.73	12.35 (0.96)	11.34–13.65	11.90–12.66
Tibial transverse shaft diameter at nutrient foramen	11.99 (2.24)	6.87–14.62	11.24–13.47	14.41 (3.08)	12.03–18.68	12.23–15.65
Tibial sagittal midshaft diameter	10.50 (1.71)	6.42–12.07	9.95–11.83	11.34 (1.31)	9.94–12.78	10.43–12.24
Tibial transverse midshaft diameter	10.03 (1.96)	5.48–12.58	9.24–11.01	10.34 (1.70)	8.81–12.71	9.31–10.94
Minimum shaft circumference	35.50 (7.38)	19.00–47.00	33.00–38.75	35.50 (3.42)	32.00–40.00	33.50–37.00
Tibial midshaft shape index	95.34 (9.73)	83.07–107.69	86.07–103.35	90.85 (5.94)	85.52–99.45	87.93–92.00

Age class 3	Males			Females		
	Media (*SD*)	Min–max	1Qrt–3Qrt	Media (*SD*)	Min–max	1Qrt–3Qrt
Maximum tibial length	159.00 (23.43)	144.00–186.00	145.50–166.50	157.43 (23.74)	111.00–176.00	150.50–174.50
Tibial sagittal shaft diameter at nutrient foramen	15.90 (0.43)	15.64–16.40	15.65–16.03	14.50 (2.58)	10.58–17.39	13.09–16.33
Tibial transverse shaft diameter at nutrient foramen	14.90 (1.21)	14.14–16.29	14.20–15.28	13.47 (1.88)	10.70–15.37	12.36–14.58
Tibial sagittal midshaft diameter	13.67 (1.02)	12.57–14.59	13.22–14.23	12.75 (2.03)	9.36–14.74	11.47–14.26
Tibial transverse midshaft diameter	11.88 (0.57)	11.37–12.50	11.57–12.13	11.67 (1.57)	9.03–13.19	10.96–12.77
Minimum shaft circumference	41.33 (1.53)	40.00–43.00	40.50–42.00	39.57 (5.16)	30.00–44.00	38.00–43.00
Tibial midshaft shape index	87.36 (10.49)	80.60–99.44	40.50–42.00	92.24 (9.37)	80.94–110.28	87.42–94.30

*Note*: Age class 1 = 0–1 years of age; age class 2 = 1.1–3 years of age; age class 3 = 3.1–6 years of age.

**TABLE 6:** Descriptive statistics (mean, standard deviation, max–min values, interquartile ranges) for the fibula, considering the whole sample

	Mean (*SD*)	Min–max	1Qrt–3Qrt
Maximum fibular length	90.36 (35.18)	38.18–182.00	57.32–110.50
Fibular maximum diameter at midshaft	4.78 (1.56)	2.19–8.24	3.36–6.02
Fibular minimum diameter at midshaft	3.72 (1.36)	1.68–7.56	2.43–4.57
Circumference at midshaft	18.21 (3.34)	10.00–27.00	17.50–19.00
Sagittal diameter at neck	4.41 (1.01)	1.96–6.81	3.69–4.91
Transverse diameter at neck	4.27 (0.95)	2.31–6.82	3.55–4.62
Maximum diameter at neck	4.70 (1.04)	2.45–7.02	3.89–5.17
Minimum diameter at neck	3.93 (0.86)	1.99–6.48	3.36–4.40
Circumference at neck	15.58 (1.93)	12.00–22.00	15.00–15.79
Fibular neck shape index	98.16 (9.07)	70.41–129.11	96.01–98.79
Sagittal diameter at midshaft	4.34 (1.39)	1.95–8.33	3.42–4.76
Transverse diameter at midshaft	4.35 (1.33)	2.06–8.41	3.28–4.56
Fibular midshaft shape index	103.67 (16.54)	63.64–164.78	96.37–103.67
Distance from neck to STS	61.54 (21.24)	30.56–127.26	40.72–64.90
Distance from neck to ILA	70.91 (26.09)	31.26–153.79	44.40–76.53
ILA length	9.92 (2.96)	3.94–19.50	8.45–9.98
STS length	19.32 (7.06)	7.44–44.81	15.14–21.79
Distance from STS to ILA	10.40 (5.20)	1.61–33.22	7.58–10.40
STS–ILA index	14.23 (3.60)	3.67–24.21	13.00–14.35
STS index	20.95 (3.47)	13.20–37.16	19.67–20.96
ILA index	12.08 (2.34)	4.76–17.70	11.40–12.96

Abbreviations: ILA, interosseous ligament attachment; STS, subcutaneous triangular surface.

**TABLE 7:** Descriptive statistics (mean, standard deviation, max–min values, interquartile ranges) for the fibula, considering age classes and sex groups

Age class 1	Males			Females		
	Mean (*SD*)	Min–max	1Qrt–3Qrt	Media (*SD*)	Min–max	1Qrt–3Qrt
Maximum fibular length	68.07 (18.79)	38.18–96.00	54.89–90.36	77.70 (19.55)	51.41–113.20	56.94–90.36
Fibular maximum diameter at midshaft	3.80 (0.99)	2.19–5.98	3.08–4.79	4.23 (0.98)	2.79–6.04	3.23–4.79
Fibular minimum diameter at midshaft	2.88 (0.83)	1.68–4.40	2.20–3.72	3.28 (0.91)	1.98–4.62	2.35–3.79
Circumference at midshaft	16.96 (2.16)	12.00–19.00	15.00–18.21	16.66 (3.06)	10.00–21.00	15.50–18.21
Sagittal diameter at neck	3.86 (0.75)	1.96–4.81	3.46–4.41	4.07 (0.72)	2.99–5.51	3.32–4.41
Transverse diameter at neck	3.75 (0.64)	2.31–4.41	3.12–4.27	3.93 (0.58)	2.74–4.57	3.46–4.27
Maximum diameter at neck	4.12 (0.74)	2.45–4.84	3.51–4.71	4.37 (0.71)	3.30–5.76	3.73–4.71
Minimum diameter at neck	3.47 (0.62)	1.99–4.39	2.85–3.93	3.62 (0.55)	2.70–4.40	3.05–3.93
Circumference at neck	14.92 (1.10)	12.00–16.00	13.00–15.58	14.48 (1.56)	12.00–15.58	13.00–15.58
Fibular neck shape index	98.64 (8.12)	70.41–117.86	98.16–100.68	97.81 (6.87)	82.94–115.14	97.59–98.16
Sagittal diameter at midshaft	3.66 (0.88)	2.05–4.76	2.90–4.34	3.70 (1.02)	1.95–4.76	2.58–4.34
Transverse diameter at midshaft	3.64 (0.89)	2.06–4.72	2.98–4.35	3.96 (0.81)	2.57–5.35	3.48–4.35
Fibular midshaft shape index	102.66 (16.86)	63.64–137.02	100.49–103.67	112.77 (18.02)	96.62–164.78	103.67–116.98
Distance from neck to STS	50.63 (12.60)	30.56–62.88	37.77–61.54	53.03 (11.90)	34.61–61.54	39.11–61.54
Distance from neck to ILA	57.04 (15.89)	31.26–70.91	41.89–70.91	60.94 (14.87)	37.96–72.49	42.38–70.91
ILA length	8.69 (1.96)	3.94–11.42	7.19–9.92	8.78 (1.89)	4.43–9.95	7.93–9.92
STS length	15.65 (4.47)	7.44–19.55	11.14–19.33	16.55 (4.76)	8.38–23.07	11.31–19.33
Distance from STS to ILA	7.87 (2.96)	1.61–10.40	4.98–10.40	9.02 (2.96)	4.40–15.01	6.15–10.40
STS–ILA index	13.40 (3.53)	3.67–23.67	12.22–14.23	14.28 (2.01)	11.59–20.71	14.00–14.23
STS index	20.42 (2.29)	13.38–25.03	20.36–20.96	20.32 (1.91)	15.68–24.52	19.41–20.96
ILA index	12.74 (1.80)	7.48–17.70	12.95–12.96	12.33 (1.49)	8.29–14.34	12.37–12.96

Age class 2	Males			Females		
	Mean (*SD*)	Min–Max	1Qrt–3Qrt	Media (*SD*)	Min–max	1Qrt–3Qrt
Maximum fibular length	105.56 (20.71)	63.94–137.00	94.01–111.00	117.12 (4.87)	112.00–122.00	113.50–120.88
Fibular maximum diameter at midshaft	5.76 (1.20)	3.34–7.53	4.95–6.50	6.21 (0.58)	5.45–6.83	5.95–6.53
Fibular minimum diameter at midshaft	4.41 (0.93)	2.47–6.07	4.17–4.61	4.94 (0.60)	4.38–5.78	4.65–5.09
Circumference at midshaft	19.72 (3.04)	15.00–26.00	18.41–20.00	19.00 (2.16)	16.00–21.00	18.25–20.25
Sagittal diameter at neck	5.12 (0.83)	3.45–6.27	4.56–5.63	5.15 (0.56)	4.41–5.65	4.88–5.55
Transverse diameter at neck	4.89 (0.86)	3.15–6.03	4.37–5.56	5.40 (0.97)	4.27–6.41	4.79–6.08
Maximum diameter at neck	5.32 (0.89)	3.50–6.49	4.79–5.90	5.75 (0.79)	4.71–6.44	5.37–6.31
Minimum diameter at neck	4.60 (0.80)	2.95–5.36	4.05–5.25	4.80 (0.60)	3.97–5.34	4.69–5.09
Circumference at neck	16.51 (1.75)	13.00–19.00	15.69–18.00	17.39 (1.46)	15.58–19.00	16.64–18.25
Fibular neck shape index	95.80 (7.08)	81.02–106.35	92.38–98.16	105.21 (16.00)	89.86–127.18	96.08–111.04
Sagittal diameter at midshaft	5.15 (1.02)	2.90–6.19	4.52–5.80	5.24 (0.84)	4.34–6.14	4.64–5.84
Transverse diameter at midshaft	4.63 (0.76)	3.02–5.84	4.35–5.07	5.67 (0.95)	4.35–6.63	5.45–6.08
Fibular midshaft shape index	91.91 (10.48)	76.88–104.14	83.54–101.88	109.51 (13.04)	94.79–124.47	101.14–117.75
Distance from neck to STS	68.52 (14.78)	34.75–91.88	62.89–76.05	74.65 (9.73)	61.54–83.92	70.51–80.73
Distance from neck to ILA	81.20 (16.54)	45.18–103.95	73.32–85.80	88.91 (13.13)	70.91–99.80	83.34–98.05
ILA length	12.40 (3.45)	7.40–18.42	9.95–14.72	10.48(2.42)	7.68–13.55	9.36–11.45
STS length	24.77 (5.53)	19.22–36.99	20.04–27.07	24.26 (5.15)	19.32–31.05	20.89–26.71
Distance from STS to ILA	13.01 (4.74)	8.84–24.33	10.40–14.78	14.67 (3.17)	10.40–17.98	13.57–16.26
STS–ILA index	16.22 (5.20)	10.33–24.21	12.14–19.59	16.27 (1.77)	14.23–18.45	15.34–17.15
STS index	23.55 (3.40)	19.97–30.06	20.98–24.57	21.62 (2.69)	19.13–25.45	20.50–22.08
ILA index	12.21 (2.46)	7.05–15.67	11.54–13.34	9.96 (2.65)	6.86–12.96	8.41–11.57

Age class 3	Males			Females		
	Media (*SD*)	Min–max	1Qrt–3Qrt	Media (*SD*)	Min–max	1Qrt–3Qrt
Maximum fibular length	153.08 (25.04)	138.25–182.00	138.62–160.50	145.90 (34.35)	90.36–174.00	128.00–169.50
Fibular maximum diameter at midshaft	7.33 (0.48)	6.78–7.70	7.14–7.60	6.75 (1.31)	4.79–8.24	5.93–7.75
Fibular minimum diameter at midshaft	6.57 (1.06)	5.45–7.56	6.07–7.13	5.28 (1.03)	3.72–6.31	4.59–6.05
Circumference at midshaft	24.00 (4.36)	19.00–27.00	22.50–26.50	21.60 (3.17)	16.00–24.00	20.60–23.50
Sagittal diameter at neck	6.48 (0.39)	6.05–6.81	6.31–6.69	5.04 (0.91)	4.41–6.81	4.41–5.38
Transverse diameter at neck	5.93 (0.83)	5.18–6.82	5.49–6.31	4.92 (0.75)	4.27–6.04	4.27–5.43
Maximum diameter at neck	6.75 (0.39)	6.31–7.02	6.62–6.97	5.50 (0.87)	4.71–6.90	4.70–6.06
Minimum diameter at neck	5.34 (1.05)	4.41–6.48	4.77–5.80	4.46 (0.71)	3.93–5.93	3.93–4.53
Circumference at neck	19.67 (2.52)	17.00–22.00	18.50–21.00	16.54 (2.06)	15.00–21.00	15.58–16.50
Fibular neck shape index	91.53 (10.63)	85.17–103.81	85.40–94.72	99.14 (13.84)	88.69–129.11	90.84–98.16
Sagittal diameter at midshaft	7.37 (1.11)	6.16–8.33	6.89–7.98	5.62 (1.36)	4.34–7.57	4.34–6.52
Transverse diameter at midshaft	6.89 (0.96)	6.23–7.99	6.34–7.22	5.89 (1.70)	4.35–8.41	4.35–7.18
Fibular midshaft shape index	93.90 (8.43)	84.65–101.14	90.29–98.53	105.74 (13.42)	91.37–132.57	99.52–107.38
Distance from neck to STS	104.40 (11.91)	96.24–118.07	97.57–108.48	89.15 (28.00)	61.54–127.26	61.54–109.67
Distance from neck to ILA	122.45 (27.14)	106.37–153.79	106.79–130.50	102.65 (33.05)	74.78–150.52	70.91–126.78
ILA length	13.43 (5.54)	8.66–19.50	10.39–15.81	12.14 (2.82)	9.92–15.78	9.92–14.70
STS length	30.91 (12.08)	22.90–44.81	23.96–34.92	24.91 (7.07)	19.32–38.65	19.33–27.41
Distance from STS to ILA	17.02 (14.04)	8.39–33.22	8.92–21.34	14.81 (5.61)	10.40–24.81	10.40–18.03
STS–ILA index	12.77 (7.66)	7.89–21.60	8.36–15.21	14.16 (1.48)	11.47–16.48	14.00–14.47
STS index	19.73 (4.30)	16.56–24.62	17.28–21.31	20.99 (7.71)	13.20–37.16	16.85–20.96
ILA index	9.19 (4.65)	4.76–14.03	6.77–11.40	11.08 (3.20)	6.59–15.12	8.47–12.96

*Note*: Age class 1 = 0–1 years of age; age class 2 = 1.1–3 years of age; age class 3 = 3.1–6 years of age.

Abbreviations: ILA, interosseous ligament attachment; STS, subcutaneous triangular surface.

### 3.1 Correlation with age

**FIGURE 4:**
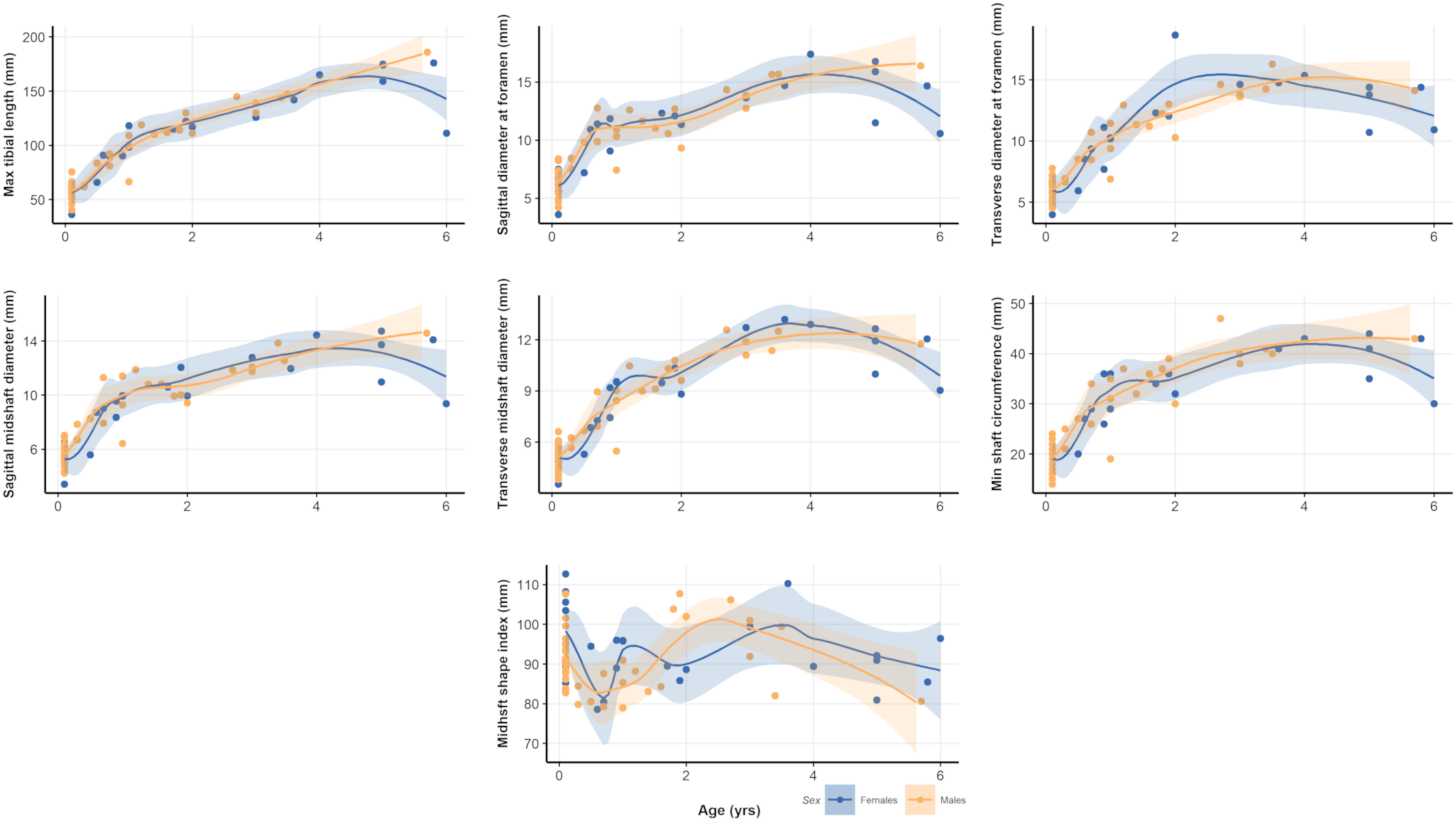
Scatter plots and LOESS‐fitted curves for tibial measurements and age, with 95% confidence intervals for females (in blue) and males (in yellow). Please refer to the online version of this article for color interpretation

**FIGURE 5:**
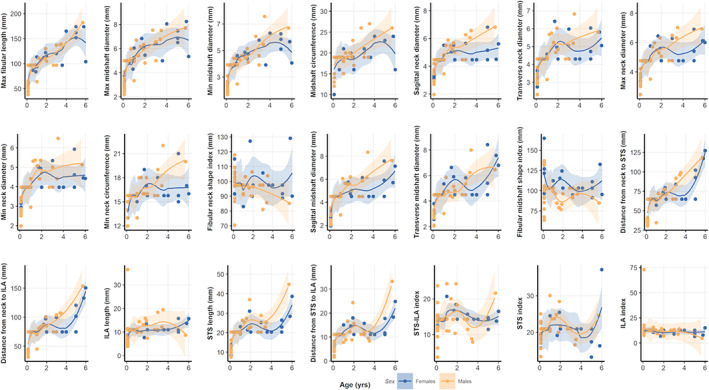
Scatter plots and LOESS‐fitted curves for fibular measurements and age, with 95% confidence intervals for females (in blue) and males (in yellow). Please refer to the online version of this article for color interpretation

All variables significantly and positively correlate with age, except for tibial and fibular indices, which appear to remain constant as age progresses. For the tibia, all linear models have *r* ^2^ values above 0.6, validating the good performance of the linear model, except for tibial midshaft shape index. For the fibula, *r* ^2^ are generally low (Table 8). Males and females display slightly different growth patterns, as highlighted by sex differences among linear models. In general, for both tibia and fibula, males show higher beta coefficient (Figures S3 and S4). LOESS fitted curves, tracing ontogenetic trajectories of males and females separately, are displayed in Figures 4 and 5. Both tibial and fibular measurements (indices excepted) show a growth pattern that is best represented by a nonlinear increase with age progression, with consistent rapid increase at earlier age and subsequent plateau after approximately the age of 4.

**TABLE 8:** Correlation between each variable and age for tibia and fibula

Tibia	Rho^a^	*r* ^2^ ^b^
Maximum tibial length	0.93***	0.82***
Tibial sagittal shaft diameter at nutrient foramen	0.89***	0.66*
Tibial transverse shaft diameter at nutrient foramen	0.89***	0.62*
Tibial sagittal midshaft diameter	0.9***	0.69*
Tibial transverse midshaft diameter	0.9***	0.69*
Minimum shaft circumference	0.88***	0.64*
Tibial midshaft shape index	−0.05	−0.15
*Fibula*		
Maximum fibular length	0.86***	0.71**
Fibular maximum diameter at midshaft	0.85***	0.59**
Fibular minimum diameter at midshaft	0.85***	0.59**
Circumference at midshaft	0.62***	0.38
Sagittal diameter at neck	0.75***	0.39
Transverse diameter at neck	0.75***	0.37
Maximum diameter at neck	0.76***	0.44
Minimum diameter at neck	0.75***	0.33
Circumference at neck	0.67*	0.3
Fibular neck shape index	−0.23	−0.01
Sagittal diameter at midshaft	0.77*	0.49*
Transverse diameter at midshaft	0.75***	0.5*
Fibular midshaft shape index	−0.22	0
Distance from neck to STS	0.75***	0.63*
Distance from neck to ILA	0.77***	0.62*
ILA length	0.63**	0.26
STS length	0.76***	0.45
Distance from STS to ILA	0.66**	0.41
STS–ILA index	0.18	0
STS index	0.16	0
ILA index	−0.18	0.11

Abbreviations: ILA, interosseous ligament attachment; STS, subcutaneous triangular surface.

^a^ *p* < 0.05 (*), *p* < 0.01 (**), and *p* < 0.001 (***), Spearman's rank correlation.

^b^ *p* < 0.05 (*), *p* < 0.01 (**), and *p* < 0.001 (***), Linear model goodness‐of‐fit.

### 3.2 Age classes and sex comparisons

Kruskal–Wallis test revealed for the tibia a significant difference among age classes for all variables, except for the midshaft shape index (Table 9). Dunn *post‐hoc* test showed significant differences (*p* < 0.05) between individuals within age class 1 and the other age classes. No significant difference was found between individuals within age class 2 and age class 3. Sagittal and transverse diameters at nutrient foramen and at midshaft in age class 1 were significantly lower than those of individuals in age class 2 and 3. Despite midshaft shape index did not differ significantly among age classes, it remained well below the value of 100 (indicating subcircular shape) for both sexes. It decreased with age among males, following the slight increase of sagittal diameters, while it slightly increased in females (Figure S5). No sex‐related significant difference was found considering all age classes together. In addition, when each age class is evaluated separately, no sex‐related intra‐class difference was detected.

**TABLE 9:** Comparisons for tibial measurements by sex and age classes

	Sex	Age class	Dunn post hoc		
	*p‐*Value^a^	*p‐*Value^a^		Age class 2	Age class 3
Maximum tibial length	°	***	Age class 1	***	***
			Age class 2		NS
Tibial sagittal shaft diameter at nutrient foramen	°	***	Age class 1	***	***
			Age class 2		NS
Tibial transverse shaft diameter at nutrient foramen	°	***	Age class 1	***	***
			Age class 2		NS
Tibial sagittal midshaft diameter	NS	***	Age class 1	***	***
			Age class 2		NS
Tibial transverse midshaft diameter	°	***	Age class 1	***	***
			Age class 2		NS
Minimum shaft circumference	NS	***	Age class 1	***	***
			Age class 2		NS
Tibial midshaft shape index	NS	NS			

*Note*: Age class 1 = 0–1 years of age; age class 2 = 1.1–3 years of age; age class 3 = 3.1–6 years of age.

Abbreviation: NS, nonsignificant result.

^a^ Kruskal—Wallis test.

^°^ 0.05 < *p* < 0.10.

*** *p* < 0.001.

Concerning the fibula, the Kruskal–Wallis test suggested a significant difference among age classes for all fibular variables, except for fibular neck and midshaft shape indices, STS‐interosseous tibiofibular ligament attachment (ILA) index and ILA index (Table 10). Dunn *post‐hoc* test showed significant differences (*p* < 0.05) in pairwise comparisons when individuals in the age class 1 are compared with the other age classes. No significant differences were found in the comparison among individual in age class 2 and age class 3, apart from the STS index. Sagittal and transverse diameters at midshaft in age class 1 were significantly lower than those of individuals in age class 2 and 3. A decrease of the midshaft shape index (i.e., subcircular shape with relative larger sagittal diameter) with age was also observed in males (Table 7), though the difference among age classes never reached significance (Table 10). The same pattern was observed also for females, though values remained above 100 for all age classes (relatively larger mediolateral diameter). Sagittal and transverse diameters at neck in age class 1 were significantly lower than those of individual in age class 2 and 3. Fibular neck shape index did not differ significantly among age classes. However, the fibular neck shape index (always lower than 100 indicating subcircular shape with relative larger sagittal diameter) decreased with age in males, while it increased in females. In particular, females of age class 2 have values above 100, indicating larger mediolateral neck diameter compared to the sagittal one (Figure S6). The only sex‐related significant differences found when considering all age classes together were for fibular midshaft index and the relative distance from STS to ILA. As already observed for the tibia, when each age class was evaluated separately, no sex‐related intra‐class differences were found.

**TABLE 10:** Comparisons for fibular measurements by sex and age classes

	Sex	Age class	Dunn post hoc		
	*p*‐Value^a^	*p*‐Value^a^		Age class 2	Age class 3
Maximum fibular length	°	***	Age class 1	***	***
			Age class 2		NS
Fibular maximum diameter at midshaft	°	***	Age class 1	***	***
			Age class 2		NS
Fibular minimum diameter at midshaft	°	***	Age class 1	***	***
			Age class 2		NS
Circumference at midshaft	NS	***	Age class 1	**	***
			Age class 2		NS
Sagittal diameter at neck	NS	***	Age class 1	***	***
			Age class 2		NS
Transverse diameter at neck	NS	***	Age class 1	***	***
			Age class 2		NS
Maximum diameter at neck	NS	***	Age class 1	***	***
			Age class 2		NS
Minimum diameter at neck	NS	***	Age class 1	***	***
			Age class 2		NS
Circumference at neck	NS	***	Age class 1	***	***
			Age class 2		NS
Fibular neck shape index	NS	NS			
Sagittal diameter at midshaft	NS	***	Age class 1	***	***
			Age class 2		NS
Transverse diameter at midshaft	°	***	Age class 1	**	***
			Age class 2		NS
Fibular midshaft shape index	**	°			
Distance from neck to STS	NS	***	Age class 1	***	***
			Age class 2		NS
Distance from neck to ILA	NS	***	Age class 1	***	***
			Age class 2		NS
ILA length	NS	***	Age class 1	**	**
			Age class 2		NS
STS length	NS	***	Age class 1	***	***
			Age class 2		NS
Distance from STS to ILA	**	***	Age class 1	***	**
			Age class 2		NS
STS–ILA index	NS	NS			
STS index	NS	**	Age class 1	**	NS
			Age class 2		**
ILA index	NS	NS			

*Note*: Age class 1 = 0–1 years of age; age class 2 = 1.1–3 years of age; age class 3 = 3.1–6 years of age.

Abbreviations: ILA, interosseous ligament attachment; NS, nonsignificant result; STS, subcutaneous triangular surface.

^°^ 0.05 < *p* < 0.10.

** *p* < 0.01.

*** *p* < 0.001.

^a^ Kruskal—Wallis test.

### 3.3 Correlation and covariation among tibia and fibula

Spearman's correlation between the set of homologous measurements is presented in Table 11. Rho values for linear measurements and circumference are >0.5, indicating that these measures on the tibia positively correlate with their counterpart on the fibula. Midshaft shape indices of the two bones are not strongly correlated.

**TABLE 11:** Spearman's correlation between tibial and fibular homologous measurements

Measures^a^	Spearman correlation
	Rho^b^
Tibial and fibular maximal lengths (T1, F1)	0.87***
Tibial and fibular sagittal midshaft diameters (T4, F11)	0.68***
Tibial and fibular transverse midshaft diameters (T5, F12)	0.66***
Tibial and fibular midshaft circumferences (T6, F4)	0.66***
Tibial and fibular midshaft shape indices (T7, F13)	−0.13

^a^ See Tables 2 and 3 for abbreviation explanation.

^b^ *p* < 0.05 (*), *p* < 0.01 (**), and *p* < 0.001 (***), Spearman's rank correlation.

### 3.4 Principal component analysis

**FIGURE 6:**
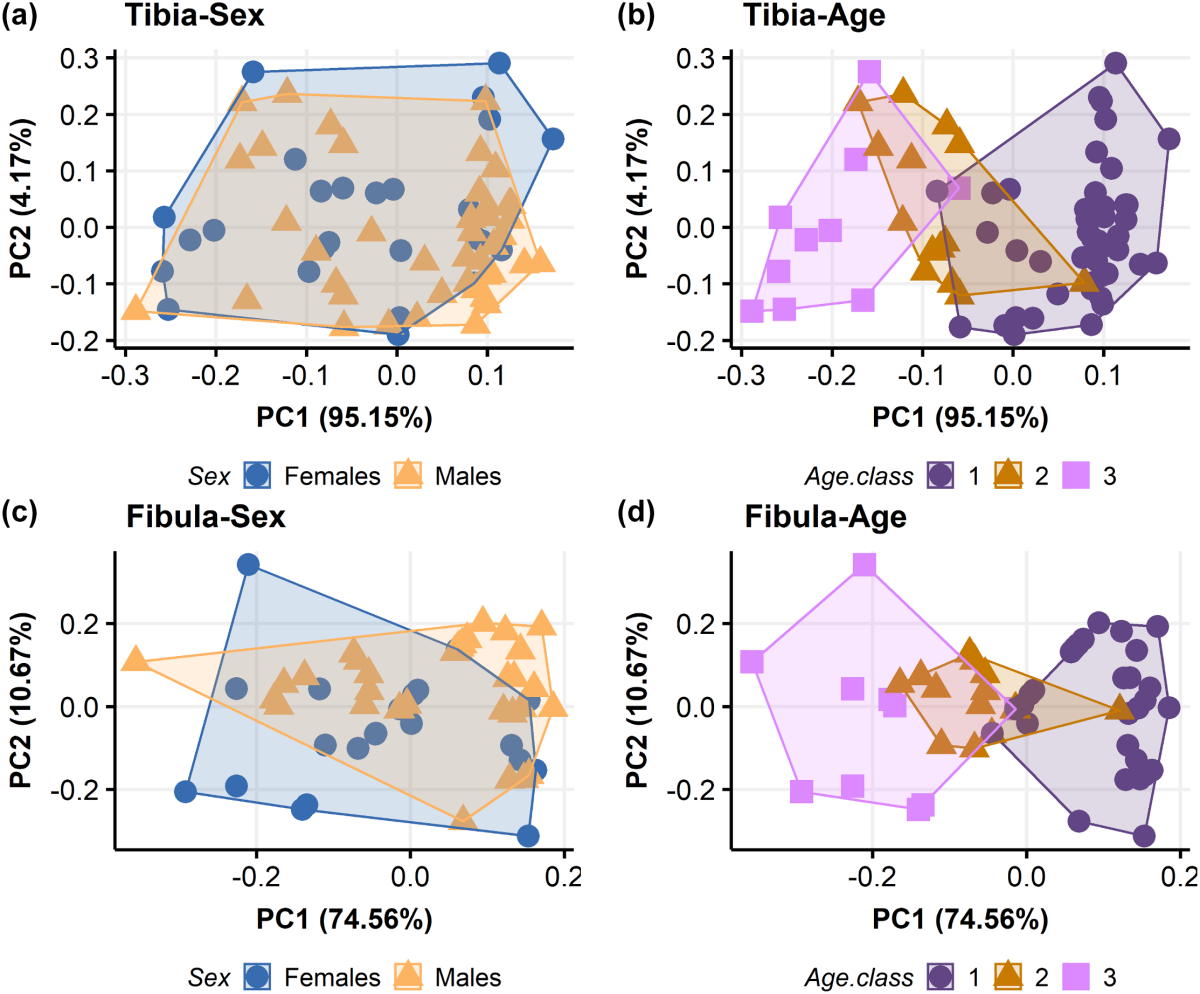
Principal component analysis plot visualizing principal component (PC) scores for tibial (top row) and fibular (bottom row) metric measurements in relation to sex (a and c) and age classes (b and d). Age class 1 = 0–1 years of age; age class 2 = 1.1–3 years of age; age class 3 = 3.1–6 years of age. Please refer to the online version of this article for color interpretation

PCA plots with PC1 and PC2 for tibial measurements in relation to sex and age classes are displayed in Figure 6a,b. PC1 explains 95.2% of variance, while PC2 explains 4.2% of variance. PC3 explains only 0.5% of total variance. PC1 is driven by maximum tibial length (loading: −0.96), while PC2 is driven by tibial midshaft shape index (loading: 0.99). PC3 is driven by minimum shaft circumference (loading: 0.84). No other variable contributes to the first three PCs. While consistent overlap is present among sexes (Figure 6a), a separation among age classes is observed along PC1 (Figure 6b). A clear distinction is present between age class 1 and age class 3, while age class 2 overlaps with the other two age classes. Maximum tibial length is therefore the variable that mainly separate age classes. No separation among groups is present along PC2, neither according to sex nor to age classes. The same trend is noted when PC2 and PC3 are plotted, but age classes tend to separate slightly along PC3 (Figure S7a,b).

PCA plots with PC1 and PC2 for fibular measurements in relation to sex and age classes are displayed in Figure 6c,d, respectively. PC1 explains 74.6% of variance, while PC2 explains 10.7% of total variation. PC3 accounts for 8.2% of variance. Both PC1 and PC2 distributions are driven by maximum fibular lengths (loadings: −0.71 and −0.51, respectively). PC1 is also loaded by the distance from fibular neck to ILA and STS (loadings: 0.52 and 0.43, respectively), while PC2 distribution is driven by fibular midshaft shape index (loading: −0.69). PC3 is also driven by fibular midshaft shape index (loading: −0.67). No other variable contributes to the first three PCs. While a high degree of overlap is present among sexes (Figure 6c), a clear separation is observed between age class 1 and age class 3. Age class 2 overlaps with both the other classes but mainly with age class 1 (Figure 6d). Maximum fibular length is the variable that mainly separate age classes. No separation among groups is present along PC2, neither according to sex nor to age classes. The same trend is noted when PC2 and PC3 are plotted (Figure S7c,d).

## 4 DISCUSSION

The present study provides quantitative data on bone linear and geometric measurements of tibia and fibula diaphyses for a sample of subadult individuals and their possible link to functional and developmental patterns in relation to sex, age, and mechanical loading. The results provide further insight into the metric characterization of long bone diaphyses during growth and expand our understanding of the timing of bone growth in the human leg. We hypothesized a significant metric variation in tibial and fibular diameters and no relevant sex differences. Our results partially corroborate our hypotheses: though no significant difference was found among age classes for tibial and fibular shape indices, we could observe a shift from a subcircular outline of the tibial and fibular diaphyses in younger individuals toward more elliptical outline with anteroposteriorly oriented major axis in older individuals at midshaft, with the exception of the fibula for females, which retain mediolaterally oriented proportions at midshaft. The same pattern was found at fibular neck in males but not in females. Further, our results suggest that no relevant sex differences are present, though unique growth trajectories have been noticed for males and females as age progresses.

### 4.1 Tibial and fibular shape and size traditional morphometrics measurements

Our results show a dynamic relationship between the developmental process of linear growth and the reshaping of the proximal and midshaft diameters with increasing age in children. The tibial diaphyseal proximal third and midshaft changes from a subcircular, symmetric outline, in age class 1 and 2, to a more anteroposteriorly oriented outline in age class 3, especially in males. The same pattern is recognizable, despite some differences, at fibular midshaft, where sagittal and transverse diameters are almost equal in individuals of age class 1. While, among older males, fibular sagittal diameters progressively increase respect to transverse diameters, older females have larger transverse midshaft diameters respect to sagittal ones in age class 2 and 3 and retain a mediolaterally oriented outline among all age classes (Tables 4, 5, 6, 7; Figures S5 and S6). Fibular neck sagittal and transverse diameters, on the contrary, do not follow the same pattern: while in males we observe a shift from a subcircular shape, following the slight increase of sagittal diameters, females have neck shape index values around and above 100, following the greater increase of the transverse neck diameter compared to the sagittal one. Our results on the tibial midshaft are consistent with previous research on tibial CSG that describe how during growth tibial diaphysis changes from a uniform rounded shape along the whole diaphysis to an asymmetric, anteroposteriorly elongated cross section (Gosman et al., 2013; Hubbell et al., 2011). Other studies (Swan et al., 2020) found the same pattern of greater degree of circularity at the proximal femur between 6 months and 1 year, similarly to what Cowgill et al. (2010) and Cowgill and Johnston (2018) found in the femur of a young walkers which are reinforced mediolaterally at midshaft. The results of the present study on the fibula of children suggest that this pattern may be common to all lower limb long bones.

Our analyses also show that tibial and fibular sagittal and transverse diameters at midshaft are closely related. This is also true for tibial and fibular maximal length growth, which both increase with age and significantly correlate with each other, as expected in the case of tibiofibular normal development (Beals & Skyhar, 1984; Ogden, 1979, 1984). Indeed, the absence of a similar growth pace of tibial and fibular diaphyses has been linked to fibular growth alterations (i.e., fibular hypoplasia or hyperplasia) that may indicate the occurrence of pathological developmental defects, possibly due to either congenital or acquired neuromuscular disorders (e.g., poliomyelitis, arthrogryposis, achondroplasia, spondyloepiphyseal dysplasia) or osteomyelitis (Beals & Skyhar, 1984; Ogden, 1979) or traumatic events such as ankle fractures (Kärrholm et al., 1984).

### 4.2 Tibial and fibular sexual dimorphism

In agreement with previous studies on tibial length of subadult individuals between 2 and 12 year of age (Cardoso et al., 2014) and on proximal and distal tibial epiphyseal breadths in individuals under 15 years of age (López‐Costas et al., 2012), our results show no sex‐related differences when males and females are compared within each age class, or in our PCA. The only significant differences between males and females were found for the fibular midshaft index and the relative distance from STS to ILA when all age classes were considered together.

The differences we observed among sexes in the fibula of children analyzed in the present study add to the several pieces of evidence that indicate some degree of sexual dimorphism in early childhood. We are not able at present to formulate a precise explanation for the fibular midshaft index and the relative distance from STS to ILA difference we found between males and females. However, this result adds to the observed relationship of the distal part of the fibula to locomotion (Marchi, 2015a) and suggests the need for further studies on biomechanics and anatomy of this region of the leg during ontogeny.

Even though little to no statistically significant differences have been found between males and females, there is some subtle sex‐related variation in the distribution pattern of the mean of some measurements (Figures S5 and S6). For most measurements, males show lower means (in mm) than females in age class 1 and 2, and higher means in age class 3. Different growth trajectories for sexes also emerged in our LOESS‐fitted curves (Figures 4 and 5). Indeed, previous studies demonstrated that sexual dimorphism appears by age 4.2–5.3 years for tibial diameters and by 2.3–11.2 years for fibular diameters, and by adolescence for tibial and fibular maximal lengths (Humphrey, 1998). Coherently, Malina and Johnston (1967) also showed that males had larger tibial diaphyseal breadths than females between 6.0 and 16.0 years, and Stull et al. (2017) found sex differences in the appendicular skeleton of children between birth and 12 years of age for tibial proximal breadth. These results agree with the observed greater cortical bone plasticity in males which is influenced by greater muscle mass during ontogeny, in turn resulting in greater long bone lengths and breadths noticeable as early as mid‐ to late childhood (Arfai et al., 2002; Cabo et al., 2012; Hogler et al., 2008; Riggs et al., 2002; Schonau, 1998; Vicente‐Rodriguez et al., 2005). However, our sample size with an unbalanced number of males and females for age class 3 prevents us from establishing proper trends. On the other hand, it is worth noting that some degree of sexual dimorphism on the pelvis prior to adolescence, detectable from early childhood, was highlighted on the same sample utilized in the current study (Marino et al., 2020).

### 4.3 Tibial and fibular traditional morphometric measurements in relation to growth trajectories

Most tibial and fibular measurements show, as expected, a significant positive correlation with age except for tibial and fibular indices which remain constant as age progresses. In addition, LOESS‐fitted curves highlighted unique growth trajectories for tibial and fibular measurements (Table 8; Figures 4 and 5; Figures S3 and S4). Tibial measurements increase rapidly until 2 years of age, approximately peaking around the age of 4, and continuing a steady, though less marked, increase at older ages. Fibular measurements follow a similar pattern, even though lengths measurements increase abruptly even after the 4‐year‐old peak. Indeed, studies on leg growth in children suggested the presence of short periods of growth velocity spurts (Hermanussen et al., 1988; Hermanussen & Burmeister, 1993). Our results are also coherent with those of Butler et al. (1990) and Bock (2004), who detected multiple growth spurts over 2‐ to 3‐year intervals, likely genetically determined.

During growth, bone structural and material properties constantly mature, increasing in length—by endochondral ossification—in size—by the continuous process of formation and resorption on the periosteal and endosteal surfaces—in bone mass, and tissue density (Kontulainen et al., 2007). The process of long bone elongation and its increase in dimensions during growth is regulated by endochondral ossification and the rate of chondrocyte proliferation occurring at the level of growth plates: during infancy, the growth plate is highly and actively functioning, causing rapid bone lengthening (Kronenberg, 2003; Lui et al., 2018). The results of the PCA (Figure 6) performed in the present study, where tibial and fibular lengths majorly contribute to the variability on PC1, agree with these findings, suggesting that longitudinal bone growth is the factor that mainly differentiate children during growth. The increase of longitudinal bone growth rate itself has been previously associated to the increased level of biomechanical stress that is experienced during the acquisition of motor skills, with the amount of physical exercise differentially influencing bone length during growth (Foster, 2019; Hammond et al., 2010). It is important to notice, however, that hormonal variations and socioeconomic context may influence longitudinal growth, which in part is genetically determined, but is also subjected to the impact of nutrition and disease, affecting the correlation of bone size with chronological age (Eveleth & Tanner, 1991; Ubelaker, 2005). For instance, Pinhasi et al. (2006) found that tibial length growth was retarded among low‐status children below 4 years of age. Additionally, there is evidence that cortical thickness, influencing long bone width, in subadults varies among high and low socioeconomic conditions (Mays et al., 2009). Thus, it is possible that the age‐related variation that we observe in tibial and fibular metrics (Figures 4 and 5) is also modeled by socioeconomic conditions. Our sample comes from a more disadvantaged, urban social context, as inferred from their burial area within the Certosa Cemetery in Bologna (Italy). Future research on possible comparisons with other subadults of higher socioeconomic status may help to better elucidate patterns of long bone metric variation in relation to growth and living conditions.

### 4.4 Tibial and fibular traditional morphometric measurements in relation to the onset of bipedal walking

Our results point to a significant difference among age classes for all tibial metric variables, except for the tibial midshaft shape index, mainly evident when individuals within age class 1 are compared with the other age classes. Significant differences among age classes have been found also for most fibular variables, apart from fibular neck and midshaft shape indices, STS–ILA and ILA indices. As for the tibia, such differences are evident when age class 1 is compared with the other age classes. Differences among age classes were also evident in the PCA (Figure 6), with age class 1 and 3 clearly separating between each other and age class 2 overlapping to the other two age classes. It is possible to interpret these patterns in light of the progressive emergence of consistent toddling attempts in growing infants around the end of the first year of life. By this age, infants usually experiment with standing on lower limbs, cruising forward, at first with support and ultimately with independent toddling (Adolph et al., 1998, 2003, 2018; Bly, 1994). Specifically, by 11–12 months of age, infants experiment unsupported toddling with a flexed swing leg that externally rotates in abduction and the stance leg in line with the trunk, not extending (Bly, 1994). In this process, both plantarflexor and dorsiflexor leg muscles (m. *tibialis anterior* and m. *gastrocnemius lateralis*) contract and activate, inducing symmetric longitudinal strain on the tibia, which progressively grows (Forssberg, 1985). Moreover, as knee flexion occurs consistently in these early ambulation attempts (Burnett & Johnson, 1971; Statham & Murray, 1971), tensile strains are applied to proximal fibula (Sarma et al., 2015). Age class 2 is characterized by a mixed motor regimen, spanning from early toddling attempts toward a more mature toddling stride (Bly, 1994): the lack of clear separation between age class 2 and the other two age classes in our PCA may reflect such events. On the other end, age class 3 consists of individuals characterized by partially to fully mature walking stride, with proper heel‐strike (Zeininger et al., 2018), and therefore, as expected, neatly distinguishable especially from age class 1.

The findings of the present study provide a solid parallel to several experimental studies on lower limb bone geometry during growth. Ireland et al. (2014) found at this time (~15 months) tibial greater bone mass, cortical bone area, pericortical circumference, and polar moment of inertia of both total and cortical bone in comparison to younger children and associated such finding with the onset and the timing of unsupported walking. Consistently, Gosman et al. (2013) identified a period between 1 and 2 years old, in which the shape of the tibial diaphyseal cross sections in the proximal half of the bone shift from relatively rounded toward triangular, anteroposteriorly oriented ones (Gosman et al., 2013). The authors interpreted the results as further evidence that the onset of bipedal walking and the relative biomechanical and loading modifications associated with it significantly affect bone morphology in early childhood (Gosman & Ketcham, 2009; Ryan & Krovitz, 2006). Ruff (2003a, 2003b) interpreted early changes in the femoral and humeral strength proportions as an effect of the initiation of upright walking as femoral growth pattern presented a velocity peak at mean age of 1.4 years. Our results are consistent with those found for the femur, indicating a general pattern for the lower limb (Cowgill et al., 2010; Swan et al., 2020).

It is important to notice that the fibula, despite following a similar pattern to the tibia for breadths and lengths at midshaft during growth, and strongly correlating with its measurements, also possesses a unique ontogenetic trajectory (Figures 4 and 5; Figures S3 and S4). Given the strong correlation of the measurements of the two leg bones, this finding further elaborates on how similar biomechanical requests act and produce comparable loading necessities on both leg bones, which interacts in load transmission through the interosseous membrane (Skraba & Greenwald, 1984; Wang et al., 1996). Our findings are also in agreement with the biomechanical investigation of an ontogenetic sample (Marchi et al., 2019) which found that fibular to tibial diaphyseal rigidity might slightly decline or remain constant from childhood through early adulthood in humans.

Limitations of this study include that, despite providing solid data on tibial and fibular cortical bone periosteal surface for the whole diaphysis, traditional morphometric measurements do not offer information on endosteal surface and medullary cavity size and shape. To overcome this issue, future work, already being implemented, will involve analysis of cross‐sectional geometrical properties of the subadult tibiofibular complex. A further limitation of the present study might also concern sample subgroups size: age class 3 males include only three individuals. On the other hand, observations on descriptive statistics and the implementation of nonparametric statistical tests helped to overcome possible numerosity issues (McDonald, 2014).

## 5 CONCLUSION

In this work, we performed a traditional morphometric analysis of tibia and fibula of subadult individuals (*n* = 68) aging 0–6 years, belonging to the Human Identified Skeletal Collection of the University of Bologna (Italy), to further our understanding of tibia and fibula variation through ontogeny. Concerning our main research goal, that is, testing the morphometric signature at the onset of bipedal walking in children, we found statistically significant variations in tibial and fibular lengths and breadths. Our results suggest a trend from a subcircular outline at tibial and fibular midshaft in younger individuals toward more anteroposteriorly oriented diaphyseal outlines in older individuals, except for females' fibular indices. The same trend is observable at fibular neck for males but not for females. Such result is interpreted as the consequence of the emergence of consistent toddling attempts in growing individuals around the end of the first year of life. As expected, and despite some degree of variation, no relevant sex differences have been found among individuals, suggesting that morphometric tibial and fibula growth might become more evident and diverge between sexes only in later childhood. This further underline how possible biomechanical requirements, determining long bone shape and size, may prevail onto pre‐existing biological features.

The present study show that leg bones morphometric variation is strongly related to age. These results further increase our knowledge on human growth variation, particularly susceptible to secular trends due to differences genetical, nutritional, environmental, and health factors. Overall, our results offer an insight on the ontogenetic trajectories of tibia and fibula, considering both biological variation and biomechanical requirements of different loading regimens.

## CONFLICT OF INTEREST

The authors have no conflict of interest to declare.

## AUTHOR CONTRIBUTIONS

**Annalisa Pietrobelli**: Conceptualization; investigation; formal analysis; validation; writing—original draft; methodology; writing—review and editing. **Damiano Marchi**: Conceptualization; supervision of the research; methodology; writing—review and editing. **Maria Giovanna Belcastro**: Conceptualization; supervision of the research; project administration; writing—review and editing.

## Supporting information


**FIGURE S1** Box plots for tibial measurements by age classes, considering males and females separately. 1 = age class 1 from 0 to 1 year of age; 2 = age class 2 from 1.1 to 3 years of age; 3 = age class 3 from 3.1 to 6 years of age. Black lines are the medians, boxes are the interquartile ranges, whiskers are the nonoutlier ranges and black circles are outliers.

**FIGURE S2** Box plots for fibular measurements by Age Classes, considering males and females separately, 1 = age class 1 from 0 to 1 year of age; 2 = age class 2 from 1.1 to 3 years of age; 3 = age class 3 from 3.1 to 6 years of age. Black lines are the medians, boxes are the interquartile ranges, whiskers are the nonoutlier ranges and black circles are outliers.

**FIGURE S3** Scatter plots and linear regression models for tibial measurements and age, with 95% confidence intervals for males (in yellow) and females (in blue).

**FIGURE S4** Scatter plots and linear regression models for fibular measurements and age, with 95% confidence intervals for males (in yellow) and females (in blue).

**FIGURE S5** Connected scatterplot with tibial measurements mean comparison for males (in yellow) and females (in blue) among different age classes (circle = age class 1; square = age class 2; diamond = age class 3).

**FIGURE S6** Connected scatterplot with fibular mean comparison for males (in yellow) and females (in blue) among different age classes (circle = age class 1; square = age class 2; diamond = age class 3).

**FIGURE S7** Principal component analysis plots visualizing PC2 and PC3 for tibial (top row) and fibular (bottom row) measurements in relation to sex (a; c) and age classes (b; d). Age class 1 = 0–1 years of age; age class 2 = 1.1–3 years of age; age class 3 = 3.1–6 years of age.

## Data Availability

The data that support the findings of this study are available from the corresponding author upon request.
