# Supplementary figures and images for: The relationship between bipedalism and growth: A metric assessment in a documented modern skeletal collection (Certosa Collection, Bologna, Italy)
Source: Am J Biol Anthropol. 2021 Nov 16;177(4):669–89. doi: 10.1002/ajpa.24440 (PMC9299160; doi:10.1002/ajpa.24440)

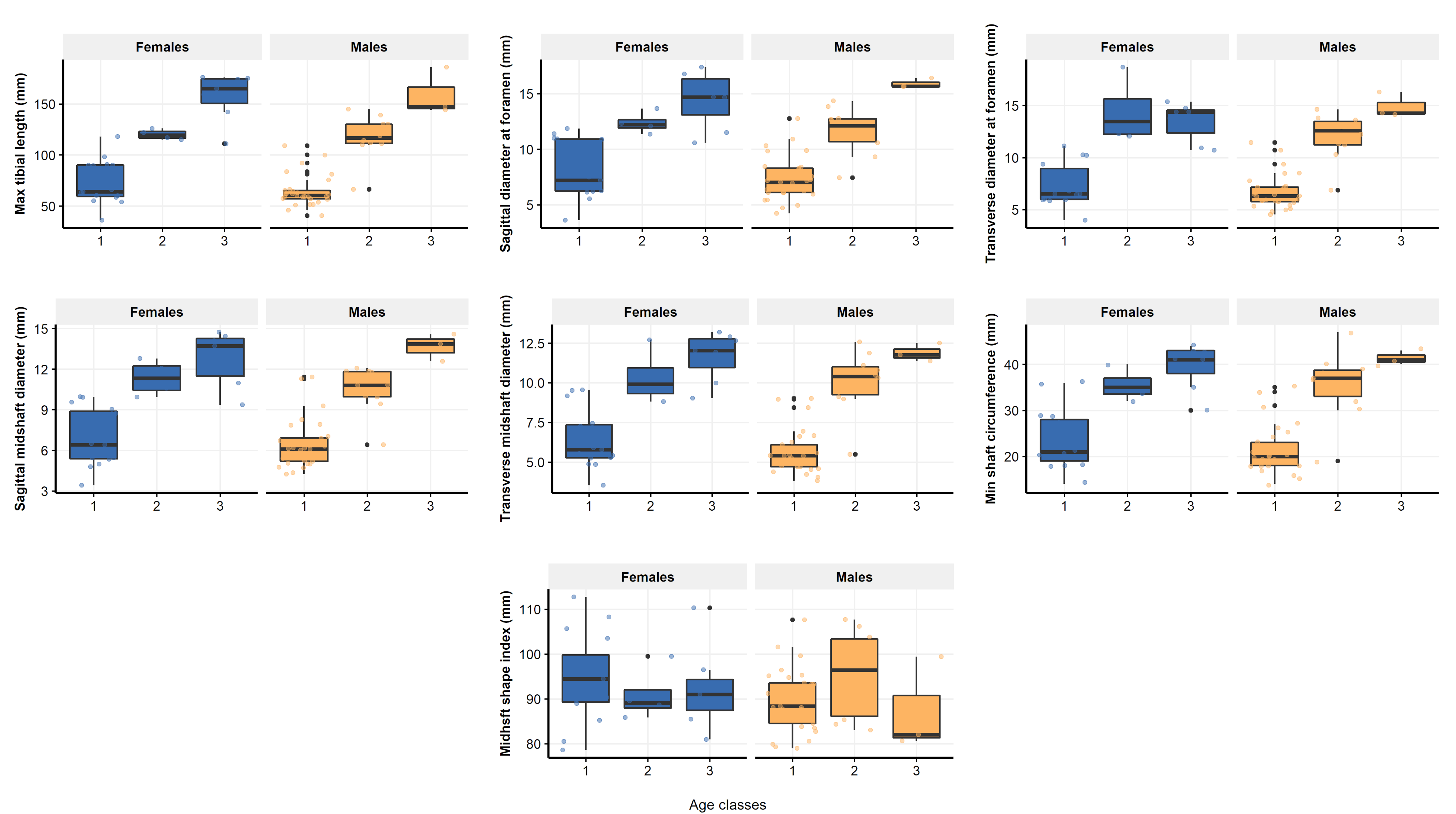

Supplement: Supplementary file 1 — FIGURE S1 Box plots for tibial measurements by age classes, considering males and females separately. 1 = age class 1 from 0 to 1 year of age; 2 = age class 2 from 1.1 to 3 years of age; 3 = age class 3 from 3.1 to 6 years of age. Black lines are the medians, boxes are the interquartile ranges, whiskers are the nonoutlier ranges and black circles are outliers. [file AJPA-177-669-s004.tiff]

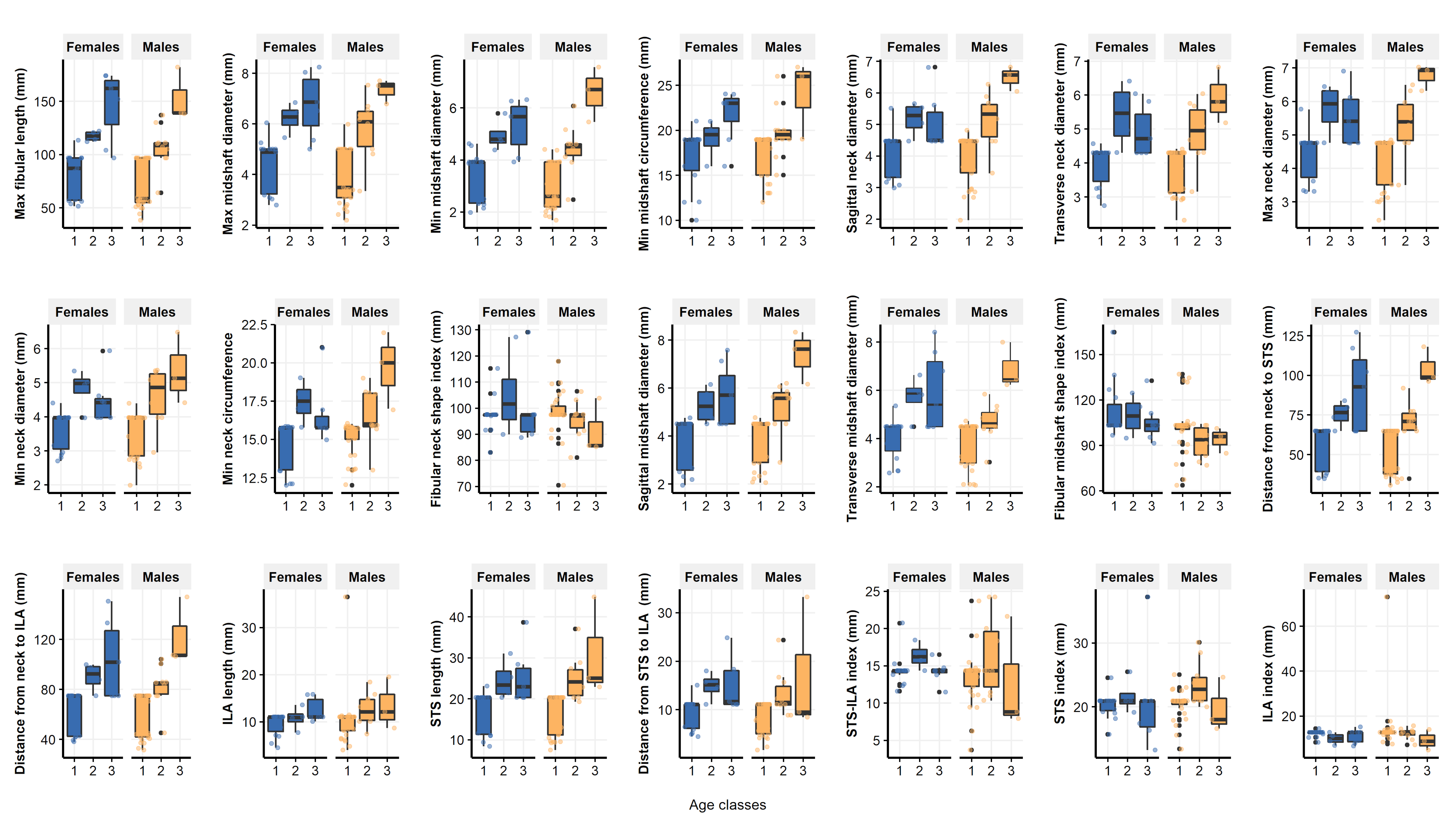

Supplement: Supplementary file 2 — FIGURE S2 Box plots for fibular measurements by Age Classes, considering males and females separately, 1 = age class 1 from 0 to 1 year of age; 2 = age class 2 from 1.1 to 3 years of age; 3 = age class 3 from 3.1 to 6 years of age. Black lines are the medians, boxes are the interquartile ranges, whiskers are the nonoutlier ranges and black circles are outliers. [file AJPA-177-669-s007.tiff]

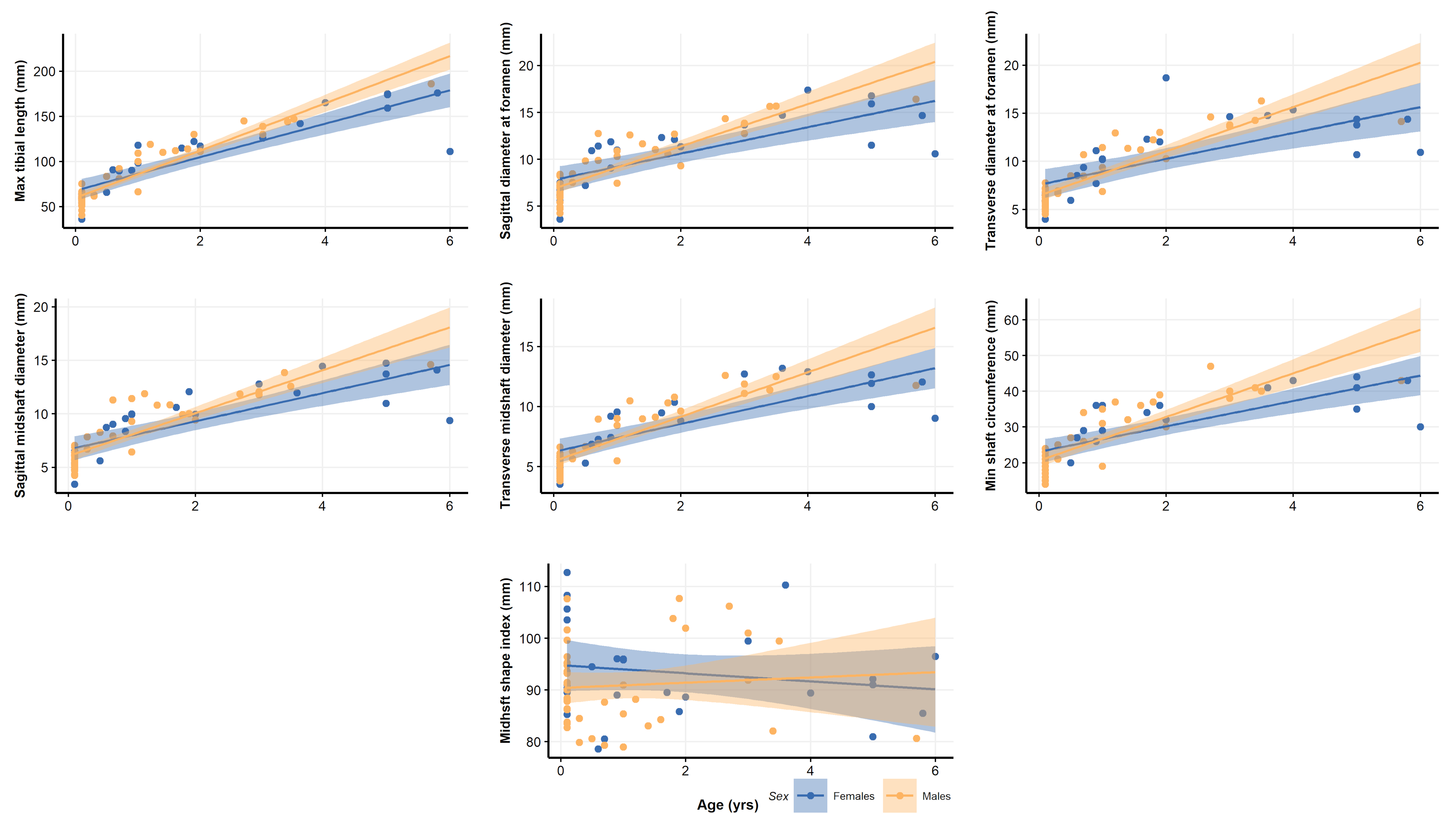

Supplement: Supplementary file 3 — FIGURE S3 Scatter plots and linear regression models for tibial measurements and age, with 95% confidence intervals for males (in yellow) and females (in blue). [file AJPA-177-669-s006.tiff]

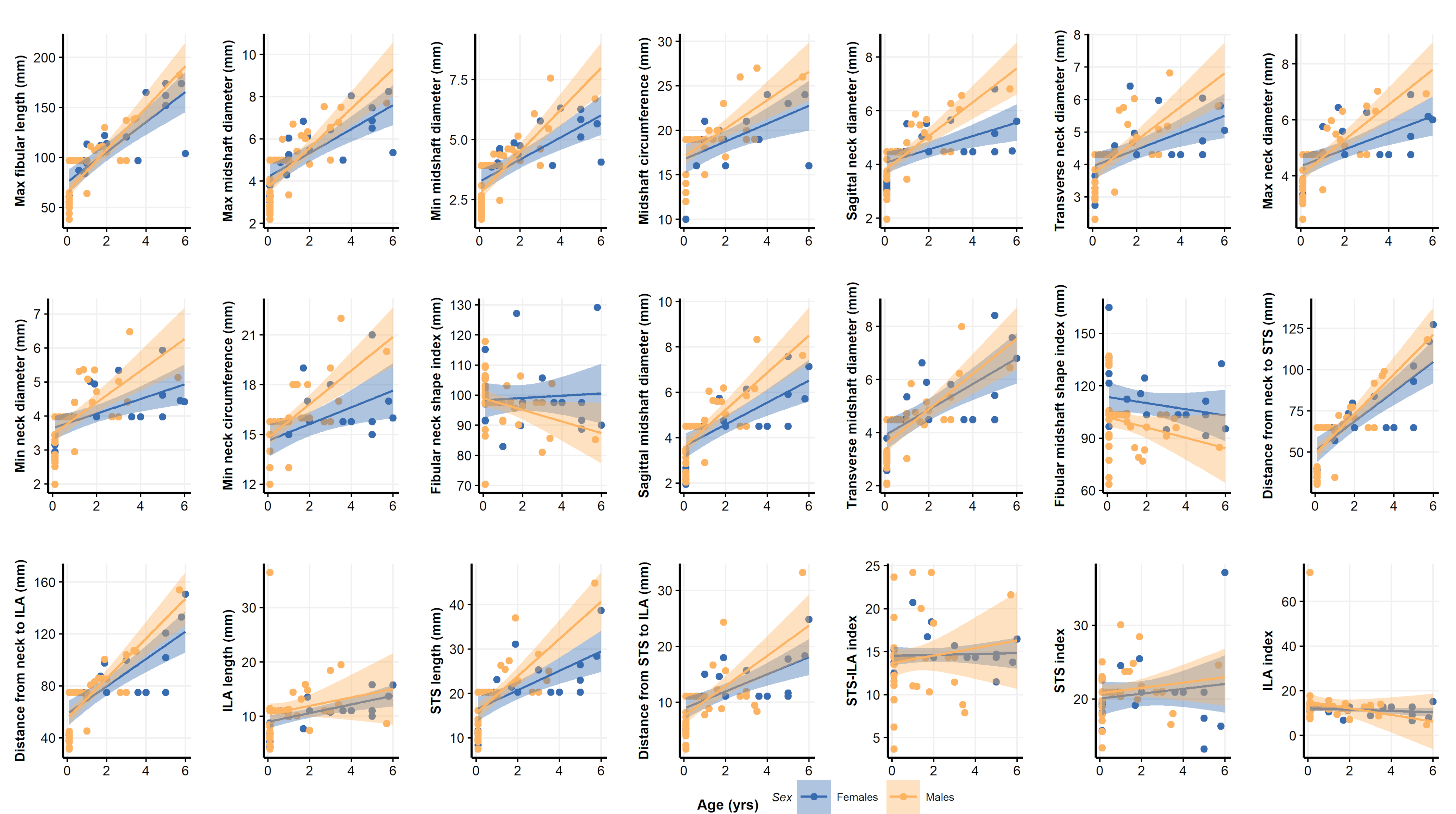

Supplement: Supplementary file 4 — FIGURE S4 Scatter plots and linear regression models for fibular measurements and age, with 95% confidence intervals for males (in yellow) and females (in blue). [file AJPA-177-669-s005.tiff]

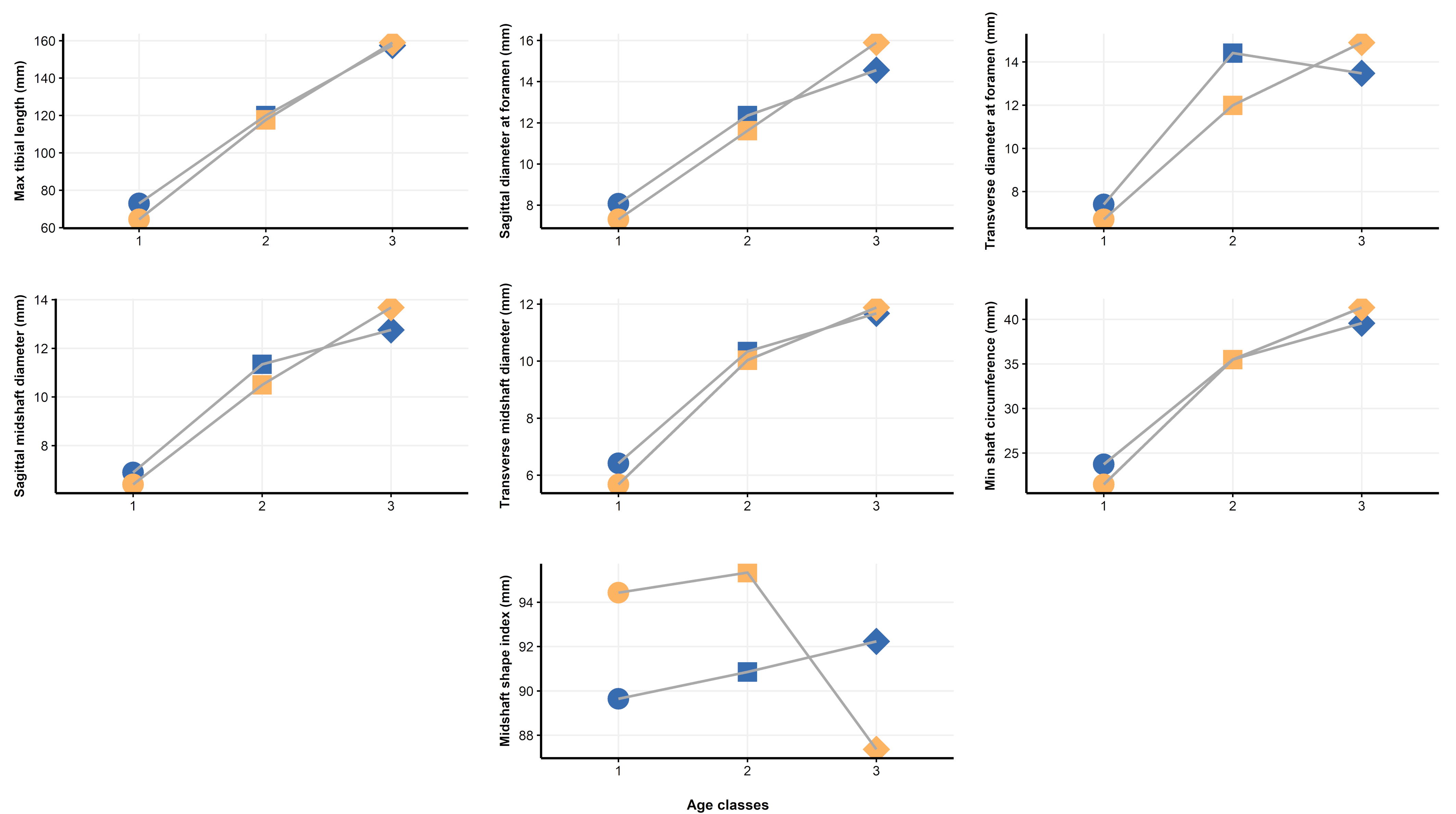

Supplement: Supplementary file 5 — FIGURE S5 Connected scatterplot with tibial measurements mean comparison for males (in yellow) and females (in blue) among different age classes (circle = age class 1; square = age class 2; diamond = age class 3). [file AJPA-177-669-s003.tiff]

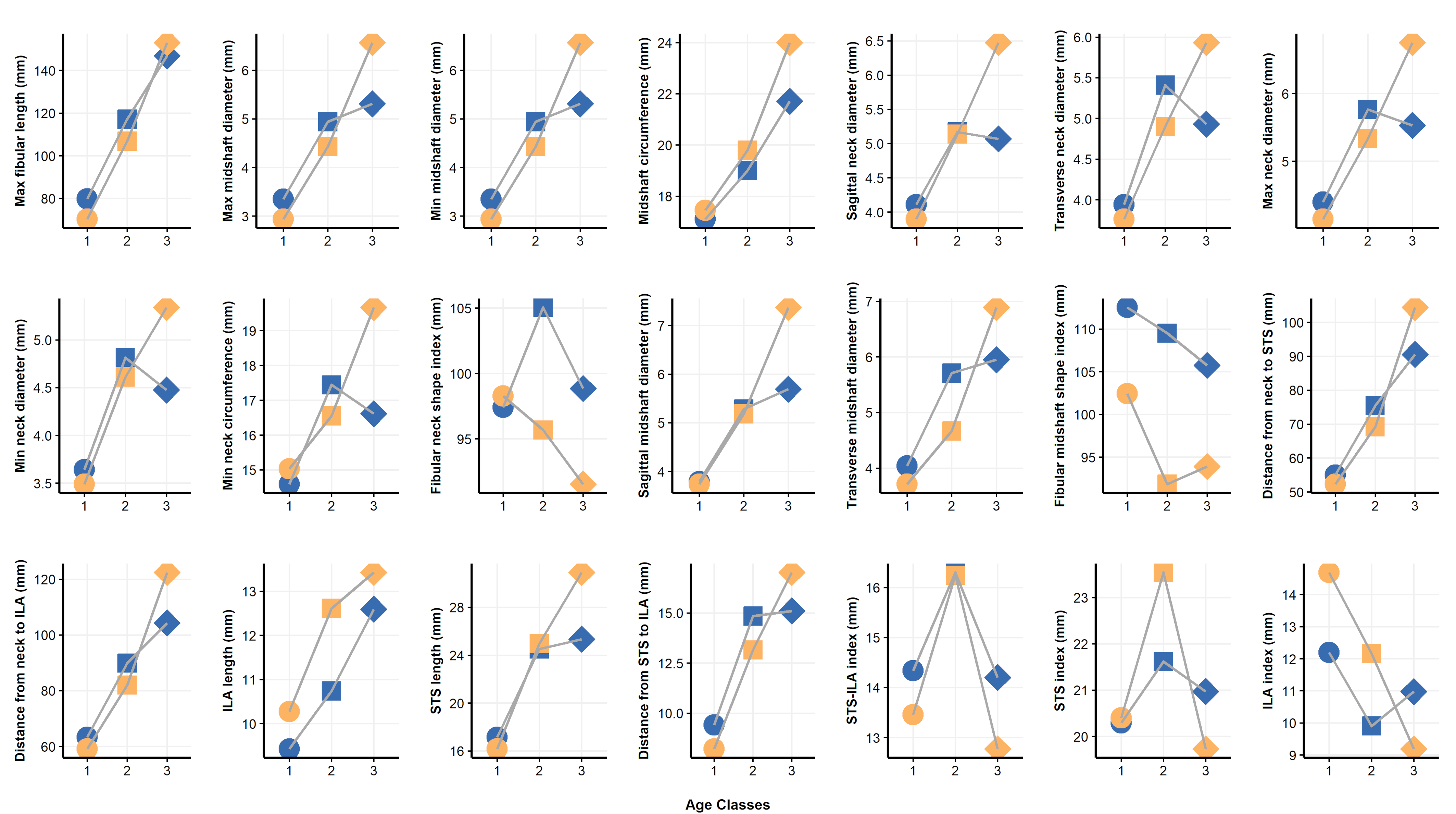

Supplement: Supplementary file 6 — FIGURE S6 Connected scatterplot with fibular mean comparison for males (in yellow) and females (in blue) among different age classes (circle = age class 1; square = age class 2; diamond = age class 3). [file AJPA-177-669-s002.tiff]

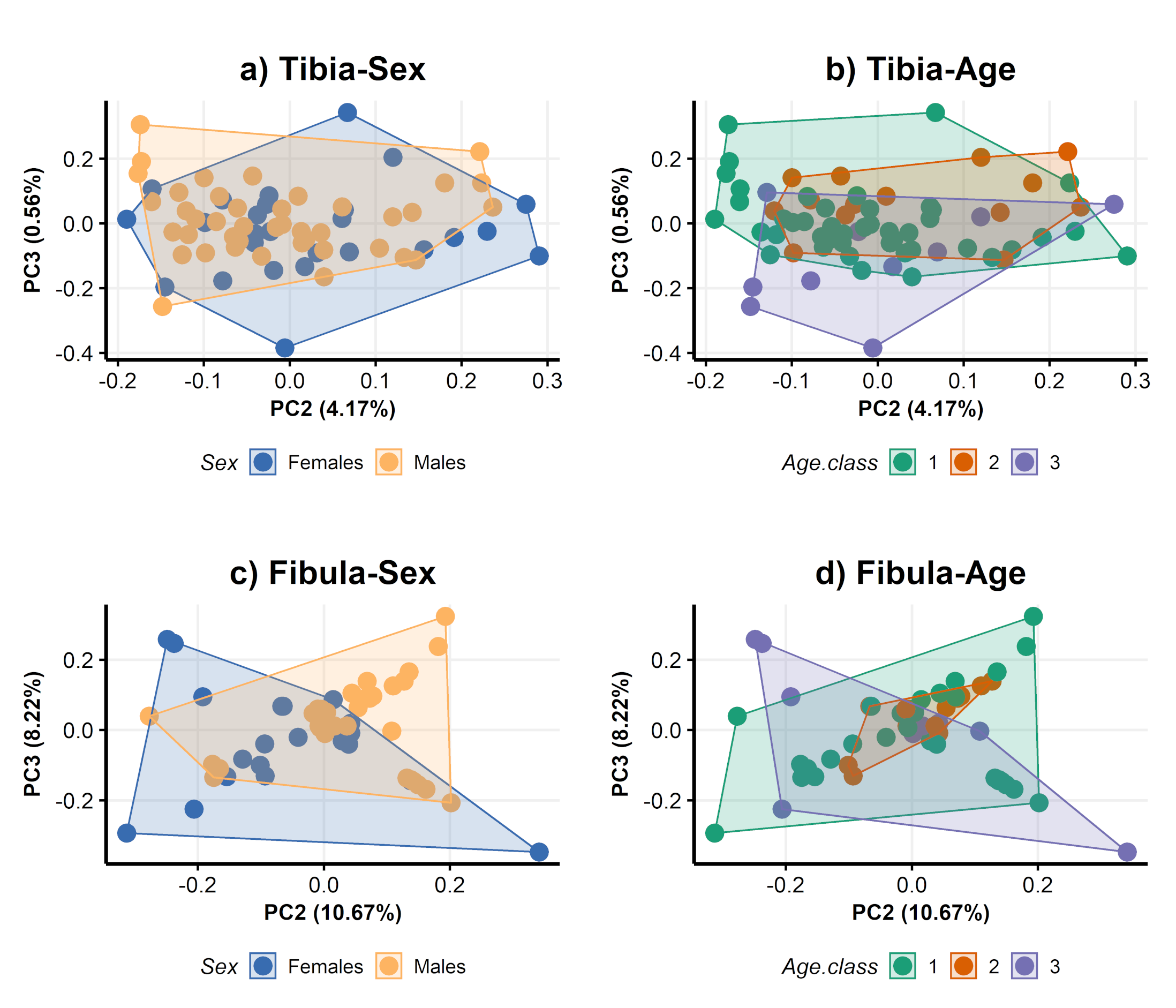

Supplement: Supplementary file 7 — FIGURE S7 Principal component analysis plots visualizing PC2 and PC3 for tibial (top row) and fibular (bottom row) measurements in relation to sex (a; c) and age classes (b; d). Age class 1 = 0–1 years of age; age class 2 = 1.1–3 years of age; age class 3 = 3.1–6 years of age. [file AJPA-177-669-s001.tiff]
